# Comparison of *In Silico* Tools for Splice-Altering Variant Prediction Using Established Spliceogenic Variants: An End-User's Point of View

**DOI:** 10.1155/2022/5265686

**Published:** 2022-10-13

**Authors:** Woori Jang, Joonhong Park, Hyojin Chae, Myungshin Kim

**Affiliations:** ^1^Department of Laboratory Medicine, College of Medicine, Inha University, Incheon, Republic of Korea; ^2^Department of Laboratory Medicine, Jeonbuk National University Medical School and Hospital, Jeonju, Republic of Korea; ^3^Department of Laboratory Medicine, College of Medicine, The Catholic University of Korea, Seoul, Republic of Korea

## Abstract

Assessing the impact of variants of unknown significance on splicing has become a critical issue and a bottleneck, especially with the widespread implementation of whole-genome or exome sequencing. Although multiple *in silico* tools are available, the interpretation and application of these tools are difficult and practical guidelines are still lacking. A streamlined decision-making process can facilitate the downstream RNA analysis in a more efficient manner. Therefore, we evaluated the performance of 8 *in silico* tools (Splice Site Finder, MaxEntScan, Splice-site prediction by neural network, GeneSplicer, Human Splicing Finder, SpliceAI, Splicing Predictions in Consensus Elements, and SpliceRover) using 114 *NF1* spliceogenic variants, experimentally validated at the mRNA level. The change in the predicted score incurred by the variant of the nearest wild-type splice site was analyzed, and for type II, III, and IV splice variants, the change in the prediction score of *de novo* or cryptic splice site was also analyzed. SpliceAI and SpliceRover, tools based on deep learning, outperformed all other tools, with AUCs of 0.972 and 0.924, respectively. For *de novo* and cryptic splice sites, SpliceAI outperformed all other tools and showed a sensitivity of 95.7% at an optimal cut-off of 0.02 score change. Our results show that deep learning algorithms, especially those of SpliceAI, are validated at a significantly higher rate than other *in silico* tools for clinically relevant *NF1* variants. This suggests that deep learning algorithms outperform traditional probabilistic approaches and classical machine learning tools in predicting the *de novo* and cryptic splice sites.

## 1. Introduction

Hereditary disorders are frequently caused by genetic variants that affect pre-mRNA splicing [1]. For genes such as *ATM* and *NF1*, the proportion of splicing variants is distinctively high, and up to 50% of the pathogenic variants result from aberrant splicing [2–4]. While most of the spliceogenic variants affect existing splice sites, exonic nucleotide variants, some of which are filtered out at the earliest bioinformatic stages as synonymous, non-synonymous or nonsense single nucleotide variants (SNVs) can also result in altered splicing [5, 6]. Moreover, whole-genome and exome sequencing is now used widely as part of routine clinical diagnostics and assessing the impact of variants of unknown significance (VUSs) on splicing has become a crucial issue and a bottleneck in clinical practice [7].

Precise pre-mRNA splicing is a complex process that relies on the coordinated interplay of various *cis*- and *trans*-acting elements [8]. The essential *cis*-acting elements are the 5′/3′ splice sites and the branchpoint sequences. Additional *cis*-elements include exonic or intronic splicing enhancers and silencers (ESEs, ISEs, ESSs, ISSs). *Trans*-acting elements include serine/arginine (SR)-rich proteins and heterogeneous nuclear ribonucleoproteins (hnRNPs). Although variants affecting the highly conserved motifs of the canonical GT-AG splice-site sequences (the first two and last two of an intron) almost invariably disrupt splicing, assessing the impact of more discrete variants involving the noncanonical sequences or deep intronic variants is a major challenge. Many *in silico* tools based on different prediction approaches are available for predicting the effect on splicing [1]. However, the application and interpretation of the output of these tools are difficult and practical guidelines are still lacking or only applicable for a few genes [9].

Neurofibromatosis type 1 (NF1), one of the most common autosomal dominant disorders, affects about 1 in 3,500 individuals in all ethnic groups. *NF1* has a high frequency of splicing variants. The *NF1* gene consists of 58 consecutive exons, which spans over 350 kb of genomic DNA. It has one of the highest mutation rates known for human genes, and the majority of mutations are scattered throughout the whole *NF1* coding sequence with no apparent mutation hotspots. Notably, 30–50% of the deleterious *NF1* mutations affect mRNA splicing, and approximately 30% of these splicing mutations are located outside the consensus splicing sequences [10–12]. The large size of the gene without any mutation predilection sites illustrates the compelling need for a streamlined decision-making process for assessing VUSs for their effect on splicing so that further downstream RNA analysis can be performed efficiently and accurately.

In this study, we compared different *in silico* prediction tools using *NF1* splice-site altering variants. Well-established databases of *NF1* splicing variants that were experimentally characterized at the genomic and mRNA level were used. Several *in silico* prediction tools were assessed and compared for their accuracy and optimal cutoffs.

## 2. Patients and Methods

### 2.1. Data Sources

All positive *NF1* splice-site altering variants from (i) two published data sources of Wimmer et al. and (ii) 43 variants identified in an NF1 patients' cohort assessed at Seoul St. Mary's Hospital (between 2011 and 2018) and Inha University of Korea were merged [13, 14]. All positive variants were experimentally validated as affecting splicing at the mRNA level. Negative splice variants were retrieved from previously reported data sets of Houdayer et al., in which the impact of the intronic variants (*BRCA1*, *BRCA2*) was experimentally validated as not affecting splicing [19]. Variant nomenclature was based on the following NCBI RefSeq accession numbers: *NF1*: NM_001042492.3, *BRCA1*: NM_007300.4, and *BRCA2*: NM_000059.4 and GRCh37 genome build. The consensus splice sites referred to in this paper are –3 to +8 at the 5′ splice site and –12 to +2 at the 3′ splice site [8]. The Institutional Review Boards of each institution approved this study.

The positive *NF1* splicing variants were classified into five categories according to Wimmer et al.: type I, classical splice-site variants leading to exon skipping; type II, cryptic exon inclusion caused by deep intronic mutations creating *de novo* splice sites; type III, exonic variants, creating *de novo* splice sites whose use results in loss of exonic sequences; type IV, activation of cryptic exonic or intronic splice sites upon canonical splice-site disruption; and type V, exonic sequence alterations causing exon skipping [13]. For the published *NF1* splicing variants, each variant was classified into five categories (Table S1A) according to the original publications [13, 14]. For the splicing variants identified from the local NF1 patients' cohort, a relevant type was assigned according to the criteria mentioned above.

### 2.2. NF1 Mutation Analysis of the Local NF1 Patients' Cohort at the Genomic DNA and RNA Levels

Genomic DNA and total RNA were extracted from the peripheral blood lymphocytes of the 43 patients using standard protocols. For stabilization of intracellular RNA during preanalytical processing of samples, PAXgene Blood RNA tubes (PreAnalytiX Qiagen/BD, Hombrechtikon, Switzerland) were used. Reverse transcription and cDNA synthesis were performed using 2 *μ*g of total RNA with Transcriptor First Strand cDNA Synthesis Kit (Roche, Mannheim, Germany) and random hexamers. PCR was performed using primers targeting the mRNA coding region of the *NF1* gene, designed to cover the whole cDNA sequence in overlapping fragments [23, 28]. The PCR products were analyzed by agarose gel electrophoresis, and all fragments were analyzed by bidirectional direct sequencing to screen altered splicing and coding region variants of *NF1*. Mutations identified by the cDNA approach were confirmed using genomic DNA sequencing with primers designed to cover the corresponding exon and adjoining introns (will be provided upon request). Sanger sequencing of PCR products was performed bidirectionally with the BigDye Terminator v3.1 Cycle Sequencing Kit (Applied Biosystems, Foster City, CA), and the products were resolved on ABI 3130XL Genetic Analyzer (Applied Biosystems). Sequences were analyzed using Sequencher (Gene Codes Corporation, Ann Arbor, MI) and were compared with the corresponding cDNA reference sequence NM_001042492.3 and gDNA reference sequence NG 009018.1.

### 2.3. In Silico Prediction Tools

A total of eight *in silico* prediction tools were assessed in this study: Splice Site Finder (SSF-like), MaxEntScan (MES), Splice-site prediction by neural network (NNSplice), GeneSplicer, Human Splicing Finder (HSF), SpliceAI, Splicing Predictions in Consensus Elements (SPiCE), and SpliceRover [6, 10, 13, 16, 17, 20, 27, 28]. The selection criteria for the choice of these eight *in silico* tools were that the tool is widely applied in routine diagnostics, has a web-based interface, can be applied to a variant in either variant or sequence format, and can predict a score for most of the variants in the dataset. SSF-like, MES, NNSplice, and GeneSplicer were analyzed using a commercial annotation software platform called Alamut Visual Plus (Interactive Biosoftware, Rouen, France, version 1.3) with the following thresholds and score ranges in brackets: SSF-like ≥0 [0–100], MES ≥0 [donor 0–12; acceptor 0–16], NNSplice ≥0 [0–1], and GeneSplicer ≥0 [donor 0–24; acceptor 0–21].

A change in the predicted score incurred by the variant at the nearest wild-type splice site (donor or acceptor) was used to evaluate each splice-site variant. For those *NF1* variants that resulted in a creation of *de novo* or activation of a cryptic splice site (such as type II, III, and IV spliceogenic variants), the change in the prediction score of the *de novo* or cryptic splice site was also analyzed. Delta scores were calculated for those *in silico* tools that provided an independent score for the wild-type and the variant [1]. For SpliceAI and SPiCE, tools that do not offer independent scores to the wild-type and the variant, the delta score of the variant supplied by the SpliceAI and SPiCE probability score, respectively, was used.

(1) $$\begin{array}{c} \text{Delta score}=\left|\frac{\text{WTscore}-\text{variant score}}{\text{Maximum score of the tool}}\right| \end{array}$$

Missing score rate, defined as the rate at which each *in silico* tool failed to identify the actual wild-type splice site and provided an output score of zero for the wild-type splice site, was estimated [15]. To evaluate the diagnostic accuracy of each *in silico* tool, a receiver operating characteristic (ROC) analysis was performed using the Analyse-it Method Validation Edition software, v5.68 (Analyse-it Software Ltd, City West Business Park, Leeds, UK). *P*-values less than 0.05 were considered statistically significant.

## 3. Results

### 3.1. Datasets

Exactly 114 unique *NF1* spliceogenic variants were assessed for their effect on splicing including 43 *NF1* spliceogenic variants identified from the local patients' cohort (Table S1A). Of the total 114 positive variants, type I, which is the classical exon skipping variant, was the most frequent (65%), followed by type IV (19%), type III (18%), type II (4%), and type V (1%). Of all positive variants, 73% (*n* = 83) were located within the consensus splice site (33 were within the 5′ consensus splice site and 50 were within the 3′ consensus splice site), and 25% (*n* = 29) were located within the canonical GT-AG splice sites.

Our negative dataset consisted of 64 intronic splice variants (29 *BRCA1*, 35 *BRCA2*) (Table S1B). None of the negative variants was located within the consensus splice site, 30 variants (47%) were located near the 3′ splice site (range: –113 to –13, median: –25.5), and 34 variants (53%) near the 5′ splice site (range: +9 to +104, median: +34.5).

### 3.2. In Silico Tools and Missing Score Rates

The missing score rates of each splice-site prediction tool (Table S2) were assessed before comparing the eight *in silico* prediction tools. Missing score rates were assessed for both the positive and negative variants. Of the *in silico* tools, GeneSplicer had the highest missing score rates (24.7%), followed by HSF (6.90%). We excluded *in silico* tools with missing score rates >10% from further analysis.

**Figure 1:**
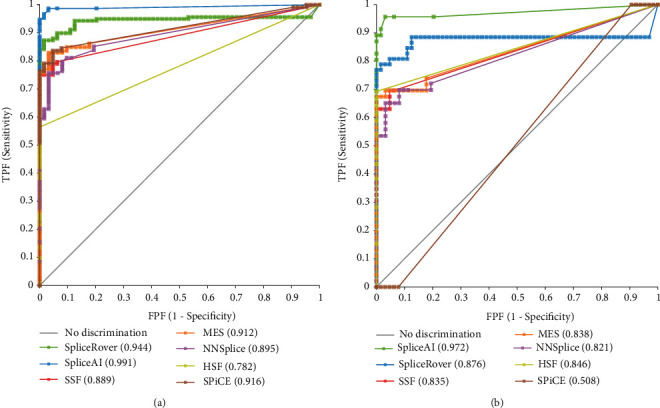
ROC curves with AUCs of the seven *in silico* tools implemented in the study (a) for all splice sites and (b) only for *de novo* or cryptic splice sites.

ROC curves with area under the curves (AUCs) of the seven *in silico* tools are shown in Figure 1 and Table 1. ROC curve plots sensitivity on the *y* axis against (1-specificity) on the *x* axis over a range of cutoff values. The ROC curve graphically displays the inherent trade-off between sensitivity and specificity by varying the choice of the cutoff. It also provides a measure known as AUC, which is an overall summary of diagnostic accuracy. An AUC of 0.5 corresponds to the accuracy achieved with random chance and an AUC of 1.0 corresponds to perfect accuracy. Most of the *in silico* tools showed high AUCs of >0.8. When prediction scores for all splice sites (wild-type splice sites, *de novo* or cryptic splice sites) were compared, SpliceAI outperformed all other tools with an AUC of 0.991. SpliceRover had a performance (AUC of 0.944) similar to that of SpliceAI, but the difference between the two *in silico* prediction tools (0.047) was statistically significant (*P* = 0.0041). SPiCE and MES performed slightly lower, with AUCs of 0.916 and 0.912, respectively. The AUCs, optimal cutoffs, sensitivity, and specificity values for *in silico* tools are summarized in Table 2.

Since most splice-site prediction tools are developed focusing on the nucleotides near the canonical and consensus splice sites, we separately assessed the prediction accuracy of *in silico* tools for *de novo* or cryptic splices sites. SpliceAI (AUC 0.972) outperformed all other tools for cryptic splice site prediction, except for SpliceRover (AUC of 0.924); the difference in prediction ability between the two tools was not statistically significant. HSF, SSF, MES, and NNSplice had slightly lower AUCs but the values were still >0.8. The majority of the *in silico* tools showed decreased AUCs when *de novo* and cryptic splice sites were assessed selectively compared to the prediction scores when wild-type splice sites were included. SPiCE showed a more significant decrease in AUCs than the other tools. Interestingly, HSF showed a higher AUC for cryptic slice site predictions than the AUCs at all splice sites (0.847 vs 0.782).

## 4. Discussion

In this study, we compared some of the most popular *in silico* prediction tools for splicing defect prediction using a well-established database of positive *NF1* variants and experimentally validated, splicing defect negative *BRCA* variants. Although the result of this study is based on positive variants of a single gene, *NF1* has a distinctively high proportion of pathogenic variants that affect splicing, spans over 350 kb, and an extensive spectrum of splicing variants have been characterized at the genomic and RNA levels. Also, the inclusion of variants not only in the canonical 5′ and 3′ splice sites and the consensus splice sites but also in the noncanonical splice site (NCSS) region allowed us to assess prediction accuracies over a greater range of variant locations. Moreover, we decided to use as our negative dataset, 64 experimentally validated intronic *BRCA* variants, all of which are located outside the consensus splice site, in nucleotide positions ranging from –13 to –113 at the 3′ splice site and from +9 to +104 at the 5′ splice site. Exploiting two different genes for evaluation of metrics of prediction tools may have introduced selection bias in this study. However, it also provided us with an opportunity to assess the prediction strengths of the “generic” *in silico* tools not only for the consensus splice sites but also for the *de novo* and cryptic splice sites and deep intronic variants.

The era of *in silico* splice prediction tool development can be categorized based on the algorithm used: the tools based on motif-based algorithms, tools based on classical machine learning, and most recently, tools based on deep learning algorithms [1]. Traditional models such as the position weight matrix (PWM) model and maximum entropy distribution (MED) approximation have been proven to be simple, understandable, and successful models for splice-site prediction [16]. Machine learning approaches have been extensively applied to newer *in silico* tools for splice-site prediction. Machine learning techniques can be further divided into classical machine learning and deep learning; the former requires preselected features for distinguishing true splice sites and false ones, while the latter automatically extracts features and optimizes a criterion for classification [1, 16]. Of the *in silico* tools assessed in this study, SpliceAI and SpliceRover are deep learning tools, NNSplice and GeneSplicer are classical machine learning tools, and SSF, MES, HSF, and SPiCE are tools that are not based on machine learning. In this study, SpliceAI and SpliceRover, two methods based on deep learning, outperformed all the other tools for included variants. SpliceAI outperformed all other tools for the prediction of *de novo* and cryptic splice sites. While most tools assessed in this study showed high AUCs for predicting the effect of variants located at canonical splice sites and consensus splice sites, the extraordinary accuracy achieved by SpliceAI was highlighted when the sensitivities of these different tools, for *de novo* or cryptic splice sites, were compared. SpliceAI showed a sensitivity of 95.7% for *de novo* or cryptic splice-site predictions at an optimal cutoff of 0.02 score change, which coincides with the suggested threshold by the developers [17]. SpliceRover showed a sensitivity of 76.9%, and the other tools showed a sensitivity of less than 70%.

Our results are consistent with those of recent studies that compared multiple *in silico* tools for genes other than *NF1*. For instance, in a study that compared 85 splice-altering variants located in the canonical splice sites or consensus splice sites, SpliceAI outperformed MES, NNSplice, SSF, and HSF, with an accuracy of 0.91 [18]. In another study that compared 213 variants located in NCSS and deep intronic (DI) regions of *ABCA4* gene and *MYBPC3* gene, SpliceAI outperformed other *in silico* prediction tools including other deep learning tools such as SpliceRover, DSSP, and MMSplice and showed the highest accuracy, positive predictive value (PPV), sensitivity, specificity, negative predictive value (NPV) and Matthews correlation coefficient (MCC) for DI variants [1]. Moreover, in one study evaluating 285 *NF1* variants, which included 26% splice variants, SpliceAI showed 94.5% sensitivity and 94.3% specificity at a cut-off value of >0.22, and performed better than the combined analysis of MES/SSF [19].

It is becoming increasingly noticeable that deep learning algorithms outperform traditional probabilistic algorithms and classical machine learning tools by incorporating long-range specificity determinants of splicing, thereby achieving significant precision [17]. Our study has shown that prediction of splice sites, especially those of SpliceAI, is validated at a significantly higher rate than other *in silico* tools using *NF1* variants. Although our data assessed a relatively small number of variants, the wide range of variant locations considered, and the inclusion of various types of aberrant splicing could make the result of this study generalizable to genes associated with aberrant splicing.

## Figures and Tables

**Table 1:** Summary of evaluation measures for 7 *in silico* tools for all splice sites.

*In silico* tools	AUC (95% CI)	Cutoff	TP	FP	TN	FN	Sensitivity	Specificity
SpliceAI	0.991 (0.980–1.002)	0.02	148	2	62	2	0.987	0.969
SpliceRover	0.944 (0.912–0.977)	0.010746	149	8	56	9	0.943	0.875
SPiCE	0.916 (0.879–0.954)	0.04424	92	3	61	18	0.836	0.953
MES	0.912 (0.878–0.946)	0.063125	126	2	60	26	0.829	0.968
NNSplice	0.895 (0.857–0.934)	0	126	12	50	22	0.851	0.806
SSF	0.889 (0.854–0.924)	0	121	4	60	31	0.796	0.937
HSF	0.782 (0.740–0.824)	0	75	0	64	58	0.564	1

Abbreviations: AUC, area under the curve; TP, true positives; FP, false positives; TN, true negatives; FN, false negatives.

**Table 2:** Summary of evaluation measures for 7 *in silico* tools only for *de novo* or cryptic splice sites.

*In silico* tools	AUC (95% CI)	Cutoff	TP	FP	TN	FN	Sensitivity	Specificity
SpliceAI	0.972 (0.937–1.008)	0.02	44	2	62	2	0.957	0.969
SpliceRover	0.924 (0.838–1.010)	0.095393	40	0	64	12	0.769	1
SPiCE	0.508 (0.457–0.559)	0.49529	0	0	64	7	0	1
MES	0.827 (0.750–0.903)	0.118125	31	0	62	15	0.674	1
NNSplice	0.818 (0.736–0.901)	0.07	28	2	60	15	0.651	0.968
SSF	0.829 (0.757–0.901)	0.0014	32	3	61	14	0.696	0.953
HSF	0.847 (0.774–0.921)	0	27	0	64	12	0.692	1

Abbreviations: AUC, area under the curve; TP, true positives; FP, false positives; TN, true negatives; FN, false negatives.

## Data Availability

The data used to support the findings of this study are available from the authors upon request.

## Supplementary Materials

Supplementary Materials Figure S1: Example of in vitro analysis of a novel variant (assessed *NF1* variant No. 32). Table S1A: the list of positive variants and their *in silico* prediction results for effect on splicing. Table S1B: the list of negative variants and their *in silico* prediction results for effect on splicing. Table S2: the missing rates of eight in silico tools
