# Supplementary material for: Comparison of In Silico Tools for Splice-Altering Variant Prediction Using Established Spliceogenic Variants: An End-User's Point of View
Source: Int J Genomics. 2022 Oct 13;2022:5265686. doi: 10.1155/2022/5265686 (PMC9584665; doi:10.1155/2022/5265686)
Supplement: Supplementary Materials — Figure S1: Example of in vitro analysis of a novel variant (assessed NF1 variant No. 32). Table S1A: the list of positive variants and their in silico prediction results for effect on splicing. Table S1B: the list of negative variants and their in silico prediction results for effect on splicing. Table S2: the missing rates of eight in silico tools [file 5265686.f1.docx]

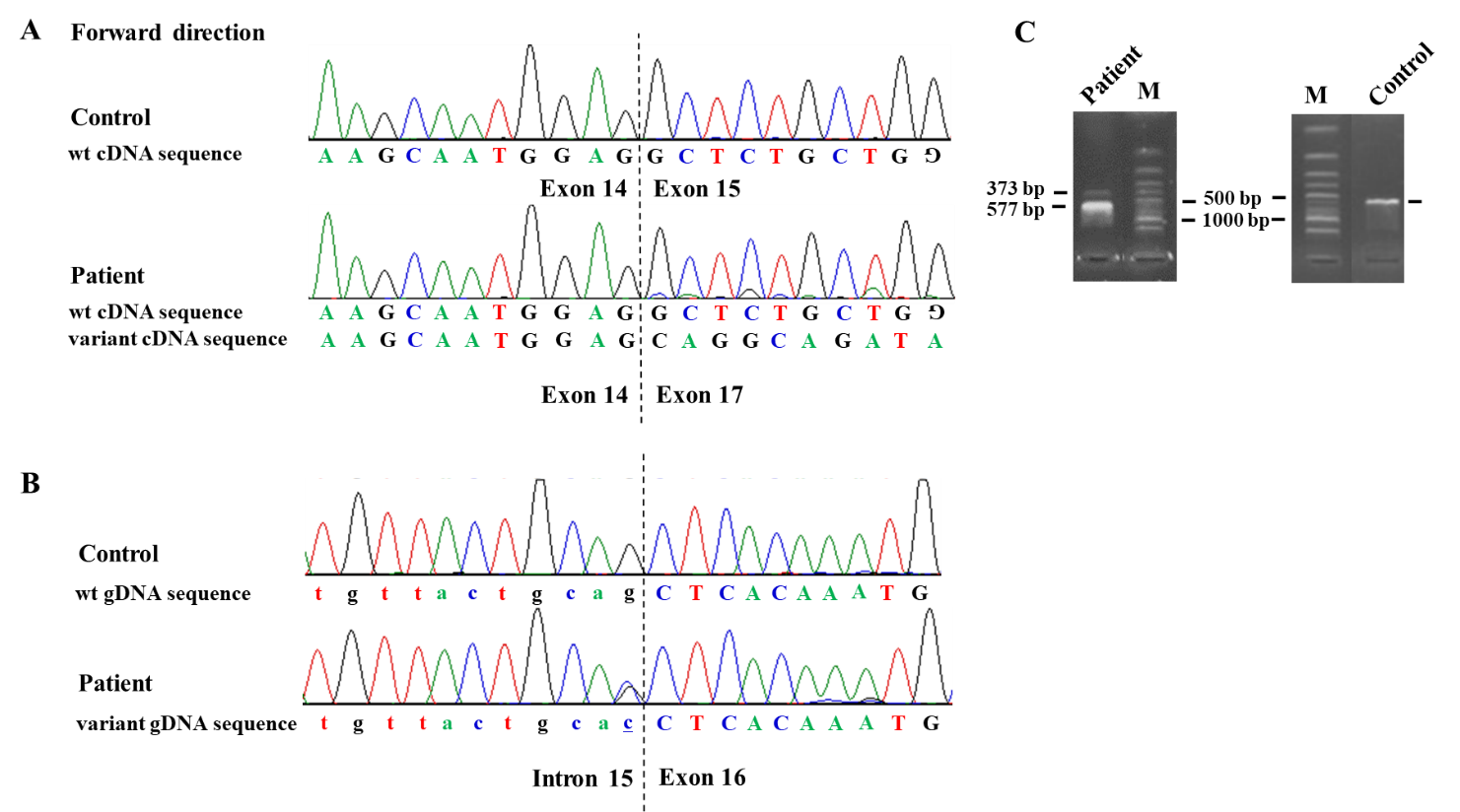


Fig. S1. Example of in vitro analysis of a novel variant. (A) Electropherogram of the complementary DNA (cDNA) sequencing of patient (assessed *NF1* variant No. 32) carrying *NF1* mutation c.1722-1G>C showed the background sequence, which is absent in the control, is derived from exon 17 indicating the presence of a small proportion of transcripts lacking exons 15 and 16. (B) Genomic DNA (gDNA) sequencing of patient. In the gDNA sequences, the nucleotide substitution is underlined. Small letters indicate the intronic sequence. (C) RT-PCR products amplified from transcripts of the patient showed one weak band (373 bp) coming from the aberrant transcripts lacking exons 15 and 16.

| Assessed NF1 Variant No. | Datasource^a^ | Mutation location | cDNA variant  (NM_001042492.2) | Effect on mRNA level | Amino Acid Change | Splicing Mutation Classification | Affected Splice Site  (N=natural, Cryptic) | located at 5' and 3' consensus site: (Cartegni et al. 2002) | wt  [SpliceRover] | mut  [SpliceRover] | delta  [SpiceRover] | SpliceAI | wt  [SSF] | mt  [SSF] | delta [SSF] | wt  [MES] | mt  [MES] | delta  [MES] | wt  [NNSplice] | mt  [NNSplice] | delta  [NNSplice] | wt  [Gene  Splicer] | mt  [Gene  Splicer] | delta  [Gene  Splicer] | wt  [HSF] | mt [HSF] | delta  [HSF] | SPiCE  probability |
| --- | --- | --- | --- | --- | --- | --- | --- | --- | --- | --- | --- | --- | --- | --- | --- | --- | --- | --- | --- | --- | --- | --- | --- | --- | --- | --- | --- | --- |
| 1 | 3 | Intron2 | c.204+1G>T | r.100_204del105 | p.Val34_Met68del | IV (5'ss) | c.204 N (Donor Site) | Y | 0.984501 | 0 | 0.984501 | 1.00 | 86.82 | 0 | 0.8682 | 10.13 | 0 | 0.63 | 0.99 | 0 | 0.99 | 1.9 | 0 | 0.08 | 91.26 | 64.12 | 0.2714 | 1 |
| 1 | 3 | Intron2 | c.204+1G>T | r.100_204del105 | p.Val34_Met68del | IV (5'ss) | c.99 N (Cryptic Donor Site) | Y | 0.048494 | 0.073205 | 0.024711 | 0.72 | 77.67 | 77.67 | 0 | 1.98 | 1.98 | 0.00 | 0.48 | 0.48 | 0.00 | 1.9 | 0 | 0.08 | ND |  | ND | ND |
| 2 | 1 | Intron2 | c.205-19T>A | r.205_288del | p.Arg69_Gly96del | I (3'ss) | c.205 N (Acceptor Site) | N | 0.996151 | 0.810456 | 0.185695 | 0.85 | 83.38 | 83.38 | 0 | 4.4 | 2.97 | 0.09 | 0.79 | 0.74 | 0.05 | 1.24 | 0 | 0.06 | 84 | 84 | 0 | 0.28293 |
| 3 | 0 | Intron2 | c.205-13T>G | r.205_288del | p.Arg69_Gly96del | I (3'ss) | c.205 N (Acceptor Site) | N | 0.996151 | 0.122532 | 0.873619 | 0.95 | 83.38 | 0 | 0.8338 | 4.4 | 0 | 0.28 | 0.79 | 0.49 | 0.30 | 1.24 | 0 | 0.06 | 81.78 | 81.78 | 0 | 1 |
| 4 | 0 | Intron3 | c.288+1G>A | r.205_288del | p.Arg69_Gly96del | I (5'ss) | c.288 N (Donor Site) | Y | 0.974029 | 0 | 0.974029 | 0.98 | 87.98 | 0 | 0.8798 | 9.65 | 0 | 0.60 | 1 | 0 | 1.00 | ND |  | ND | 92.78 | 65.64 | 0.2714 | 1 |
| 5 | 1 | Intron3 | c.289-6T>G | r.289_479del, r.288_289ins289-5_289-1 | p.Gln97fs | **I (3'ss)**, III | c.289 N (Acceptor Site) | Y | 0.998311 | 0.261311 | 0.737 | 0.65 | 84.76 | 0 | 0.8476 | 8.34 | 2.33 | 0.38 | 0.81 | 0 | 0.81 | 5.26 | 0 | 0.25 | 87 | 87 | 0 | 1 |
| 5 | 1 | Intron3 | c.289-6T>G | r.289_479del, r.288_289ins289-5_289-1 | p.Gln97fs | I (3'ss), **III** | c.289-5 (**Cryptic** Acceptor Site) | Y | 0 | 0.533484 | 0.533484 | 0.64 | 0 | 75.02 | 0.7502 | 0 | 4.95 | 0.31 | ND |  | ND | ND |  | ND | 52.86 | 80.73 | -0.2787 | ND |
| 6 | 3 | Exon4 | c.479G>C | r.289_479del191 | p.Gln97fs | I (5'ss) | c.479 N (Donor Site) | Y | 0.996066 | 0.613298 | 0.382768 | 0.92 | 90.12 | 76.81 | 0.1331 | 8.02 | 0.44 | 0.47 | 0.99 | 0.75 | 0.24 | ND |  | ND | 93.81 | 82.65 | 0.1116 | 0.99987 |
| 7 | 1 | Intron4 | c.480-8C>G | r.480_586del | p.Leu161fs | I (3'ss) | c.480 N (Acceptor Site) | Y | 0.999305 | 0.793842 | 0.205463 | 0.95 | 93.3 | 0 | 0.933 | 11.41 | 5.35 | 0.38 | 1 | 0.99 | 0.01 | 5.68 | 0 | 0.27 | 90 | 90 | 0 | 1 |
| 8 | 1 | Intron5 | c.587-14T>A | r.587_654del | p.Glu196fs | I (3'ss) | c.587 N (Acceptor Site) | N | 0.999893 | 0.958829 | 0.041064 | 0.14 | 94.11 | 0 | 0.9411 | 8.11 | 4.03 | 0.26 | 0.66 | 0 | 0.66 | 2.62 | 0 | 0.12 | 92 | 92 | 0 | 1 |
| 9 | 1 | Intron5 | c.587-12T>A | r.587_654del | p.Glu196fs | I (3'ss) | c.587 N (Acceptor Site) | Y | 0.999893 | 0.949478 | 0.050415 | 0.52 | 94.11 | 0 | 0.9411 | 8.11 | 1.3 | 0.43 | 0.66 | 0 | 0.66 | 2.62 | 0 | 0.12 | 92 | 92 | 0 | 1 |
| 10 | 0 | Intron6 | c.654+1G>C | r.587_654del | p.Glu196fs | I (5'ss) | c.654 N (Donor Site) | Y | 0.999828 | 0 | 0.999828 | 1.00 | 99.69 | 0 | 0.9969 | 11 | 0 | 0.69 | 1 | 0 | 1.00 | 1.95 | 0 | 0.08 | 99.86 | 72.72 | 0.2714 | 1 |
| 11 | 0 | Intron6 | c.654+2T>C | r.587_654del | p.Glu196Glyfs*12 | I (5'ss) | c.654 N (Donor Site) | Y | 0.999828 | 0 | 0.999828 | 0.34 | 99.69 | 99.77 | 0.0008 | 11 | 0 | 0.69 | 1 | 0 | 1.00 | 1.95 | 0 | 0.08 | 99.86 | 72.72 | 0.2714 | 0.99269 |
| 12 | 1 | Intron7 | c.731-14T>G | r.731_835del, r.730_731ins731-13_731-1 | p.Cys245_Glu279del, p.Glu244fs | **I (3'ss)**, III | c.731 N (Acceptor Site) | N | 0.999967 | 0.993145 | 0.006822 | 0.07 | 93.28 | 88.1 | 0.0518 | 8.1 | 5.18 | 0.18 | 0.55 | 0 | 0.55 | 4.57 | 0 | 0.22 | 91 | 91 | 0 | 0.75806 |
| 12 | 1 | Intron7 | c.731-14T>G | r.731_835del, r.730_731ins731-13_731-1 | p.Cys245_Glu279del, p.Glu244fs | I (3'ss), **III** | c.731-13 (**Cryptic** Acceptor Site) | N | 0 | 0.998386 | 0.998386 | 0.98 | 0 | 73.39 | 0.7339 | 0 | 5.89 | 0.37 | 0 | 0.41 | 0.41 | 0 | 2.86 | 0.14 | 51.26 | 79.13 | 0.2787 | ND |
| 13 | 0 | Intron7 | c.731-6_733del9 | r.733_741del  exon 8 new (cryptic) splice site activation | p.Cys245_Glu247del | IV (3'ss) | c.731 N (Acceptor Site) | Y | ND |  | ND | 1.00 | 93.28 | 0 | 0.9328 | 8.1 | 0 | 0.51 | 0.55 | 0 | 0.55 | 4.57 | 0 | 0.22 | Error |  | Error | 1 |
| 13 | 0 | Intron7 | c.731-6_733del9 | r.733_741del  exon 8 new (cryptic) splice site activation | p.Cys245_Glu247del | IV (3'ss) | c.740 (**Cryptic** Acceptor Site) | Y | 0.131745 | 0.999916 | 0.868171 | 0.99 | 0 | 93.28 | 0.9328 | 0 | 7.77 | 0.49 | 0 | 0.59 | 0.59 | 0 | 2.28 | 0.11 | Error |  | Error | ND |
| 14 | 0 | Intron8 | c.888+1G>A | r.731_888del158 | p.Cys245fs | I (5'ss) | c.888 N (Donor Site) | Y | 0.999669 | 0 | 0.999669 | 0.95 | 94.67 | 0 | 0.9467 | 10.51 | 0 | 0.66 | 1 | 0 | 1.00 | 4.07 | 0 | 0.17 | 97.27 | 70.13 | 0.2714 | 1 |
| 15 | 1 | Intron8 | c.889-21C>A | r.889_1062del | p.Lys297_Lys354del | I (3'ss) | c.889 N (Acceptor Site) | N | 0.962773 | 0.774518 | 0.188255 | 0.35 | 80.01 | 80.01 | 0 | 3.66 | 3.66 | 0.00 | 0.64 | 0.47 | 0.17 | ND |  | ND | 78 | 78 | 0 | 0.02686 |
| 16 | 1 | Intron8 | c.889-12T>A | r.889_1062del, r.654_655ins655-10_655-1 | p.Lys297_Lys354del; p.Ala219fs | **I (3'ss)**, III | c.889 N (Acceptor Site) | Y | 0.962773 | 0.643521 | 0.319252 | 0.86 | 80.01 | 0 | 0.8001 | 3.66 | 0 | 0.23 | 0.64 | 0 | 0.64 | ND |  | ND | ND |  | ND | 1 |
| 16 | 1 | Intron8 | c.889-12T>A | r.889_1062del, r.654_655ins655-10_655-1 | p.Lys297_Lys354del; p.Ala219fs | I (3'ss), **III** | c.1063 (**Cryptic** Acceptor Site) | Y | 0.994671 | 0.994671 | 0 | 0.00 | ND |  | ND | ND |  | ND | ND |  | ND | ND |  | ND | 48.82 | 76.68 | -0.2786 | ND |
| 17 | 3 | Exon9 | c.989C>T | r.988_1062del75 | p.Ala330_Lys354del | III | c.987 (**Cryptic** Donor Site) | N | 0 | 0.994946 | 0.994946 | 0.93 | 87.01 | 87.56 | 0.0055 | 0 | 9.72 | 0.61 | 0 | 0.99 | 0.99 | 0 | 5.49 | 0.23 | 62.64 | 89.78 | 0.2714 | 0.02686 |
| 17 | 3 | Exon9 | c.989C>T | r.988_1062del75 | p.Ala330_Lys354del | III | c.1062 (Donor Site) | N | 0.790779 | 0.625749 | 0.16503 | 0.77 | 81.67 | 81.67 | 0 | 8.92 | 8.92 | 0.00 | 0.93 | 0.93 | 0.00 | ND |  | ND | ND |  | ND | ND |
| 18 | 0 | Exon 22 - Intron 22 | c.2891_2990+85del185 | r.2851_2990del | p.Val951_Arg997del | I (5'ss) | c.2990 N (Donor Site) | Y | 0.881116 | 0 | 0.881116 | Error | 94.22 | 0 | 0.9422 | 9.88 | 0 | 0.62 | 1 | 0 | 1.00 | 3.85 | 0 | 0.16 | ND |  | ND | 0.02686 |
| 19 | 0 | Exon10 | c.1185G>C | r.1063_1185del123 | p.Asn355_Lys395del | I (5'ss) | c. 1185 N (Donor Site) | Y | 0.995215 | 0.58167 | 0.413545 | 0.60 | 90 | 76.69 | 0.1331 | 8.68 | 5.13 | 0.22 | 0.99 | 0.74 | 0.25 | 2.09 | 0 | 0.09 | 95.55 | 84.54 | 0.1101 | 0.98919 |
| 20 | 0 | Exon10 | c.1185G>A | r.1063_1185del123 | p.Asn355_Lys395del | I (5'ss) | c. 1185 N (Donor Site) | Y | 0.995215 | 0.511425 | 0.48379 | 0.62 | 90 | 77.87 | 0.1213 | 8.68 | 4.23 | 0.28 | 0.99 | 0.51 | 0.48 | 2.09 | 0 | 0.09 | 95.55 | 84.97 | 0.1058 | 0.99297 |
| 21 | 3 | Intron10 | c.1185+1G>A | r.1063_1185del123 | p.Asn355_Lys395del | I (5'ss) | c. 1185 N (Donor Site) | Y | 0.995215 | 0 | 0.995215 | 0.99 | 90 | 0 | 0.9 | 8.68 | 0 | 0.54 | 0.99 | 0 | 0.99 | 2.09 | 0 | 0.09 | 94.34 | 67.2 | 0.2714 | 1 |
| 22 | 0 | Intron11 | c.1260+1604A>G | r.1260_1261ins1260 +1605_  1261+1646 | p.Asn420_Ser421insLeuThrThr* | II | c.1260+1605 (**Cryptic** Acceptor Site) | N | 0 | 0.986031 | 0.986031 | 0.76 | 0 | 91.74 | 0.9174 | 0 | 8.26 | 0.52 | 0 | 0.92 | 0.92 | 0 | 4.21 | 0.20 | 64.27 | 92.14 | 0.2787 | 0.02686 |
| 22 | 0 | Intron11 | c.1260+1604A>G | r.1260_1261ins1260 +1605_  1261+1646 | p.Asn420_Ser421insLeuThrThr* | II | c.1260+1646 (**Cryptic** Donor Site) | N | 0.369487 | 0.43462 | 0.065133 | 0.75 | 75.42 | 75.42 | 0 | 8.56 | 8.56 | 0.00 | 0.96 | 0.96 | 0.00 | 1.56 | 1.79 | 0.01 | ND |  | ND | ND |
| 23 | 0 | Intron11 | c.1260+2T>C | r.[1260_1261ins1260+1_1261+13;  1260+2u>c] | partial retention of intron 11, probably resulting in frameshifting premature codon termination [p.(Ser421Alafs*12)] | IV (5'ss) | c.1260 N (Donor Site) | Y | 0.999195 | 0 | 0.999195 | 0.98 | 87.08 | 86.75 | 0.0033 | 8.62 | 0 | 0.54 | 0.99 | 0 | 0.99 | 0.97 | 0 | 0.04 | 89.35 | 62.21 | 0.2714 | 0.9922 |
| 23 | 0 | Intron11 | c.1260+2T>C | r.[1260_1261ins1260+1_1261+13;  1260+2u>c] | partial retention of intron 11, probably resulting in frameshifting premature codon termination [p.(Ser421Alafs*12)] | IV (5'ss) | c.1260+13 (**Cryptic** Donor Site) | Y | 0.976255 | 0.999162 | 0.022907 | 0.08 | 72.11 | 72.11 | 0 | 7.76 | 7.76 | 0.00 | 0.83 | 0.83 | 0.00 | 0 | 1.23 | 0.05 | 75.99 | 75.99 | 0 | ND |
| 24 | 0 | Intron12 | c.1393-592A>G | r.1392_1393ins1393-672_1393-597 | premature stop codon | II | c.1393-597 (**Cryptic** Donor Site) | N | 0.000396 | 0.000396 | 0 | ND | 0 | 79.35 | 0.7935 | 0.65 | 8.78 | 0.51 | 0 | 0.98 | 0.98 | 0 | 0.96 | 0.04 | 38.61 | 66.48 | 0.2787 | 0.02686 |
| 24 | 0 | Intron12 | c.1393-592A>G | r.1392_1393ins1393-672_1393-597 | premature stop codon | II | c.1393-672 (**Cryptic** Acceptor Site) | N | 0.109994 | 0.109994 | 0 | 0.90 | 81.63 | 81.63 | 0 | 6.21 | 6.21 | 0.00 | 0.59 | 0.59 | 0.00 | 3.73 | 3.73 | 0.00 | 87.1 | 87.1 | 0 | ND |
| 25 | 3 | Exon13 | c.1466A>G | r.1466_1527del62 | p.Tyr489fs | III | c.1465 (**Cryptic** Donor Site) | N | 0 | 0.979735 | 0.979735 | 0.99 | 0 | 75.42 | 0.7542 | 0 | 8.56 | 0.54 | 0 | 0.97 | 0.97 | ND |  | ND | 55.06 | 82.2 | -0.2714 | 0.02686 |
| 25 | 3 | Exon13 | c.1466A>G | r.1466_1527del62 | p.Tyr489fs | III | c.1527(Donor Site) | N | 0.952601 | 0.663083 | 0.289518 | 0.99 | 74.3 | 74.3 | 0 | 7.65 | 7.65 | 0.00 | 0.86 | 0.86 | 0.00 | 0.8 | 1.13 | 0.01375 | ND |  | ND | ND |
| 26 | 3 | Intron13 | c.1527+4_1527+7delAGTA | r.1393_1527del135 | p.Ser465_Cys509del | I (5'ss) | c.1527 N (Donor Site) | Y | 0.952601 | 0.000184 | 0.952417 | 1.00 | 74.3 | 0 | 0.743 | 7.65 | 0 | 0.48 | 0.86 | 0 | 0.86 | 0.8 | 0 | 0.03 | 81.88 | 57.71 | 0.2417 | 1 |
| 27 | 3 | Intron13 | c.1527+1159C>T | r.1527_1528ins1527+1104_  1527+1157 | premature stop codon | II | c.1527+1157 (**Cryptic** Donor Site) | N | 0 | 0.771956 | 0.771956 | ND | 79.45 | 82.85 | 0.034 | 0 | 7.66 | 0.48 | 0 | 0.92 | 0.92 | ND |  | ND | 60.18 | 87.32 | -0.2714 | 0.02686 |
| 27 | 3 | Intron13 | c.1527+1159C>T | r.1527_1528ins1527+1104_  1527+1157 | premature stop codon | II | c.1527+1104 (**Cryptic** Acceptor Site) | N | 0.952349 | 0.994397 | 0.042048 | 0.98 | 85.59 | 85.59 | 0 | 10.19 | 10.19 | 0.00 | 0.95 | 0.95 | 0.00 | 3.63 | 3.41 | 0.01 | ND |  | ND | ND |
| 28 | 0 | Exon15 | c.1642G>C | r.1642_1702del | p.Ala548_Ser574del | I (3'ss) | c.1642 N (Acceptor Site) | Y | 0.798418 | 0.476477 | 0.321941 | 0.16 | 75.81 | 70.05 | 0.0576 | 4.83 | 3.19 | 0.10 | 0.01 | 0 | 0.01 | 1.4 | 0 | 0.07 | 79.57 | 75.41 | 0.0416 | 0.86413 |
| 29 | 3 | Intron15 | c.1722-2A>G | r.1721_1722ins1722- 43_1722-1 | p.Ser574fs | IV (3'ss) | c.1722 N (Acceptor Site) | Y | 0.925503 | 0 | 0.925503 | 0.83 | 85.36 | 0 | 0.8536 | 5.54 | 0 | 0.35 | 0.23 | 0 | 0.23 | ND |  | ND | 84.06 | 56.19 | 0.2787 | 1 |
| 29 | 3 | Intron15 | c.1722-2A>G | r.1721_1722ins1722- 43_1722-1 | p.Ser574fs | IV (3'ss) | c.1722-43 (**Cryptic** Acceptor Site) |  | 0.637337 | 0.90134 | 0.264003 | 0.48 | 75.25 | 75.25 | 0 | 5.3 | 5.3 | 0.00 | 0.43 | 0.43 | 0.00 | 1.52 | 1.71 | 0.01 | ND |  | ND | ND |
| 30 | 1 | Intron15 | c.1722-26T>C | r.1721_1722ins1722-43_1722-1 | p.Ser574fs | IV | c.1722 N (Acceptor Site) | N | 0.925503 | 0.391303 | 0.5342 | 0.50 | 85.36 | 85.36 | 0 | 5.54 | 5.54 | 0.00 | 0.23 | 0.23 | 0 | ND |  | ND | ND |  | ND | 0.02686 |
| 30 | 1 | Intron15 | c.1722-26T>C | r.1721_1722ins1722-43_1722-1 | p.Ser574fs | IV | c.1722-43 (**Cryptic** Acceptor Site) | N | 0.637337 | 0.898252 | 0.260915 | 0.46 | 75.25 | 75.25 | 0 | 5.3 | 5.3 | 0.00 | 0.43 | 0.43 | 0.00 | 1.52 | 2.04 | 0.02 | ND |  | ND | ND |
| 31 | 1 | Intron15 | c.1722-11T>G | r.[1642_1845del, 1722_1845del] | p.[Ala548_Lys615del, Ser575fs] | I (3'ss) | c.1722 N (Acceptor Site) | Y | 0.925503 | 0.006049 | 0.919454 | 0.84 | 85.36 | 0 | 0.8536 | 5.54 | 0 | 0.35 | 0.23 | 0.05 | 0.18 | ND |  | ND | 84 | 84 | 0 | 1 |
| 32 | 2 | Intron15 | c.1722-1G>C | r.1642_1845del | p.Ala548_Lys615del | I (3'ss) | c.1722 N (Acceptor Site) | Y | 0.925503 | 0 | 0.925503 | 0.83 | 83.36 | 0 | 0.8336 | 5.54 | 0 | 0.35 | 0.23 | 0 | 0.23 | ND |  | ND | 84.06 | 56.19 | 0.2787 | 1 |
| 33 | 3 | Intron17 | c.2002-19_2005del23 | r.2002_2011del10 | p.Asp668fs | IV (3'ss) | c.2002 N (Acceptor Site) | P | ND |  | ND | 0.98 | 85.21 | 0 | 0.8521 | 8.12 | 0 | 0.51 | 0.72 | 0 | 0.72 | 4.36 | 0 | 0.21 | 0 | 0 | 0 | 1 |
| 33 | 3 | Intron17 | c.2002-19_2005del23 | r.2002_2011del10 | p.Asp668fs | IV (3'ss) | c.2012 N (**Cryptic** Acceptor Site) |  | 0.019133 | 0.322321 | 0.303188 | 0.00 | 0 | 79.48 | 0.7948 | 0 | 3.02 | 0.19 | 0 | 0 | 0 | ND |  | ND | 0 | 0 | 0 | ND |
| 34 | 1 | Intron18 | c.2252-13T>A | r.2252_2325del | p.Arg752fs | I (3'ss) | c.2252 N (Acceptor Site) | N | 0.991778 | 0.086341 | 0.905437 | 0.66 | 82.73 | 0 | 0.8273 | 7.31 | 1.25 | 0.38 | 0.65 | 0.17 | 0.48 | 9.12 | 0 | 0.43 | 80 | 80 | 0 | 1 |
| 35 | 0 | Exon19 | c.2325G>C | r.2252_2325del74 | p.Gly751_775Gludel | I (5'ss) | c.2325 N (Donor Site) | Y | 0.974153 | 0.172281 | 0.801872 | 0.85 | 80.99 | 67.68 | 0.1331 | 9.12 | 4.54 | 0.29 | 0.99 | 0.69 | 0.30 | 5.95 | 1.8 | 0.17 | 87.26 | 76.25 | 0.1101 | 0.99725 |
| 36 | 1 | Intron19 | c.2326-6T>G | r.2326_2409del | p.Trp777_Ala804del | I (3'ss) | c.2326 N (Acceptor Site) | Y | 0.998432 | 0.23113 | 0.767302 | 0.44 | 84.01 | 0 | 0.8401 | 7.26 | 1.51 | 0.36 | 0.97 | 0.79 | 0.18 | 2.57 | 0 | 0.12 | 85 | 85 | 0 | 1 |
| 37 | 0 | Intron19 | c.2326-1G>T | r.2326_2409del | p.Ala776_Gln803del | I (3'ss) | c.2326 N (Acceptor Site) | Y | 0.998432 | 0 | 0.998432 | 0.99 | 84.01 | 0 | 0.8401 | 7.26 | 0 | 0.45 | 0.97 | 0 | 0.97 | 2.57 | 0 | 0.12 | 85.1 | 57.23 | 0.2787 | 1 |
| 38 | 2 | Intron19 | c.2326-1G>C | r.2326_2409del | p.Ala776_Gln803del | I (3'ss) | c.2326 N (Acceptor Site) | Y | 0.998432 | 0 | 0.998432 | 0.98 | 84.01 | 0 | 0.8401 | 7.26 | 0 | 0.45 | 0.97 | 0 | 0.97 | 2.57 | 0 | 0.12 | 85.1 | 57.23 | 0.2787 | 1 |
| 39 | 0 | Intron20 | c.2409+1G>A | r.2326_2409del | p.Ala776_Gln803del | I (5'ss) | c.2409 N (Donor Site) | Y | 0.999251 | 0 | 0.999251 | 1.00 | 100 | 0 | 1 | 10.86 | 0 | 0.68 | 1 | 0 | 1.00 | 3.14 | 0 | 0.13 | 100 | 72.86 | 0.2714 | 1 |
| 40 | 3 | Exon21 | c.2709G>A | r.2707_2850del144 | p.Val903_Gln950del48 | III | c.2706 (**Cryptic** Donor Site) | N | 0.465028 | 0.793389 | 0.328361 | 0.62 | 76.65 | 81.01 | 0.0436 | 4.84 | 8.35 | 0.22 | 0.84 | 0.98 | 0.14 | 0 | 5.05 | 0.21 | 84.06 | 95.11 | 0.1105 | 0.02686 |
| 40 | 3 | Exon21 | c.2709G>A | r.2707_2850del144 | p.Val903_Gln950del48 | III | c.2850 (Donor Site) | N | 0.996559 | 0.995192 | 0.001367 | 0.10 | 82.83 | 82.83 | 0 | 9.65 | 9.65 | 0.00 | 0.92 | 0.92 | 0.00 | ND |  | ND | ND |  | ND | ND |
| 41 | 0 | Intron21 | c.2850+1G>C | r.2618_2850del233 | p.Lys874fs | I (5'ss) | c.2850 N (Donor Site) | Y | 0.996559 | 0 | 0.996559 | 0.99 | 82.83 | 0 | 0.8283 | 9.65 | 0 | 0.60 | 0.92 | 0 | 0.92 | 2.97 | 0 | 0.12 | 87.84 | 60.7 | 0.2714 | 1 |
| 42 | 1 | Intron21 | c.2851-14_2851-13insA | r.2851_2990del | p.Leu952fs | I (3'ss) | c.2851 N (Acceptor Site) | N | 0.996645 | 0.752275 | 0.24437 | 0.41 | 90.19 | 0 | 0.9019 | 11.24 | 7.71 | 0.22 | 0.99 | 0.98 | 0.01 | 8.76 | 0 | 0.42 | ND |  | ND | 0.02686 |
| 43 | 0 | Intron22 | c.2990+1G>A | r.2851_2990del | p.Val951_Arg997del | I (5'ss) | c.2990 N (Donor Site) | Y | 0.881116 | 0 | 0.881116 | 0.99 | 94.22 | 0 | 0.9422 | 9.88 | 0 | 0.62 | 1 | 0 | 1.00 | 3.85 | 0 | 0.16 | 97.07 | 69.93 | 0.2714 | 1 |
| 44 | 0, 2 | Intron22 | c.2991-1G>A | r.2991_3113del | p.Arg997_Arg1038del | I (3'ss) | c.2991 N (Acceptor Site) | Y | 0.999857 | 0 | 0.999857 | 1.00 | 81.75 | 0 | 0.8175 | 10.44 | 0 | 0.65 | 0.98 | 0 | 0.98 | 8.05 | 0 | 0.38 | 82.27 | 54.4 | 0.2787 | 1 |
| 45 | 3 | Intron22 | c.2991-1G>C | r.2991_3113del | p.Arg997_Arg1038del | I (3'ss) | c.2991 N (Acceptor Site) | Y | 0.999857 | 0 | 0.999857 | 0.93 | 81.75 | 0 | 0.8175 | 10.44 | 0 | 0.65 | 0.98 | 0 | 0.98 | 8.05 | 0 | 0.38 | 82.27 | 54.4 | 0.2787 | 1 |
| 46 | 3 | Exon23 | c.3063_3113+45del96 | r.2991_3113del123 | p.Tyr998_Arg1038del | I (5'ss) | c.3113 N (Donor Site) | P | ND |  | ND | ND | 91.51 | 0 | 0.9151 | 8.83 | 0 | 0.55 | 0.99 | 0 | 0.99 | 0.61 | 0 | 0.03 | 0 | 0 | 0 | 1 |
| 47 | 0 | Intron23 | c.3113+1G>A | r.2991_3113del | p.Arg997_Arg1038del | I (5'ss) | c.3113 N (Donor Site) | Y | 0.683985 | 0 | 0.683985 | 1.00 | 91.51 | 0 | 0.9151 | 8.83 | 0 | 0.55 | 0.99 | 0 | 0.99 | 0.61 | 0 | 0.03 | 95.65 | 68.51 | 0.2714 | 1 |
| 48 | 0 | Intron23 | c.3113+1G>T | r.2991_3113del | p.Arg997_Arg1038del | I (5'ss) | c.3113 N (Donor Site) | Y | 0.683985 | 0 | 0.683985 | 1.00 | 91.51 | 0 | 0.9151 | 8.83 | 0 | 0.55 | 0.99 | 0 | 0.99 | 0.61 | 0 | 0.03 | 95.65 | 68.51 | 0.2714 | 1 |
| 49 | 3 | Exon23 | c.3113+5G>T | r.2991_3113del | p.Tyr998_Arg1038del | I (5'ss) | c.3113 N (Donor Site) | Y | 0.683985 | 0.033083 | 0.650902 | 0.38 | 91.51 | 79.12 | 0.1239 | 8.83 | 4.02 | 0.30 | 0.99 | 0.21 | 0.78 | 0.61 | 0 | 0.03 | 95.65 | 85.95 | 0.097 | 0.99492 |
| 50 | 3 | Intron24 | c.3198-2A>T | r. 3198_3199delAG | p.Asp1067fs | IV (3'ss) | c.3198 N (Acceptor Site) | Y | 0.999002 | 0 | 0.999002 | 0.97 | 96.09 | 0 | 0.9609 | 11.78 | 0 | 0.74 | 0.99 | 0 | 0.99 | 11.11 | 0 | 0.53 | 96.94 | 69.07 | 0.2787 | 1 |
| 50 | 3 | Intron24 | c.3198-2A>T | r. 3198_3199delAG | p.Asp1067fs | IV (3'ss) | c.3200 (**Cryptic** Acceptor Site) |  | 0.326755 | 0.622593 | 0.295838 | 0.51 | 83.96 | 83.96 | 0 | 1.43 | 2.93 | 0.09 | 0.96 | 0.96 | 0.00 | 7.55 | 7.06 | 0.02 | 87 | 87 | 0 | ND |
| 51 | 3 | Exon25 | c.3278T>A | r.3275_3314del40 | p.Gly1092fs | III | c.3314 N (Donor Site) | N | 0.461612 | 0.329548 | 0.132064 | 0.43 | 71.58 | 71.58 | 0 | 1.48 | 1.48 | 0.00 | 0.13 | 0.13 | 0 | ND |  | ND | ND |  | ND | 0.02686 |
| 51 | 3 | Exon25 | c.3278T>A | r.3275_3314del40 | p.Gly1092fs | III | c.3274 (**Cryptic** Donor Site) |  | 0.209224 | 0.616169 | 0.406945 | 0.83 | 71.63 | 81.8 | 0.1017 | 2.28 | 7.61 | 0.33 | 0.05 | 0.66 | 0.61 | 0 | 1.03 | 0.04 | 81.47 | 91.77 | 0.103 | ND |
| 52 | 1 | Intron25 | c.3315-8T>G | r.3315_3496del | p.Tyr1106fs | I (3'ss) | c.3315 (Acceptor Site) | Y | 0.968052 | 0.073873 | 0.894179 | 0.53 | 94.25 | 0 | 0.9425 | 9.55 | 3.41 | 0.38 | 0.96 | 0.88 | 0.08 | 4.89 | 0 | 0.23 | 91 | 91 | 0 | 1 |
| 53 | 1, 3 | Intron25 | c.3315-3C>G | r.3315_3496del | p.Tyr1106fs | I (3'ss) | c.3315 (Acceptor Site) | Y | 0.968052 | 0.033843 | 0.934209 | 0.53 | 94.25 | 83.43 | 0.1082 | 9.55 | 0 | 0.60 | 0.96 | 0.04 | 0.92 | 4.89 | 0 | 0.23 | 91 | 91 | 0 | 0.99994 |
| 54 | 0 | Intron27 | c.3708+3_6delAAGT | r.3497_3708del | p.Gly1166_Trp1236del | I (5'ss) | c.3708 N (Donor Site) | Y | 0.968191 | 0.035704 | 0.932487 | 0.98 | 86.91 | 70.16 | 0.1675 | 10.24 | 2.26 | 0.50 | 0.99 | 0.1 | 0.89 | 2.55 | 0 | 0.11 | 91.99 | 65.64 | 0.2635 | 1 |
| 55 | 0 | Intron27 | c.3709-2A>C | r.3709_3718del | p.Asp1237fs | IV (3'ss) | c.3709 N (Acceptor Site) | Y | 0.999853 | 0 | 0.999853 | 1.00 | 95.77 | 0 | 0.9577 | 9.74 | 0 | 0.61 | 0.99 | 0 | 0.99 | 8.19 | 0 | 0.39 | 93.53 | 65.66 | 0.2787 | 1 |
| 55 | 0 | Intron27 | c.3709-2A>C | r.3709_3718del | p.Asp1237fs | IV (3'ss) | c.3719 (**Cryptic** Acceptor Site) | Y | 0.071649 | 0.994771 | 0.923122 | 0.99 | 0 | 56.29 | 0.5629 | 0 | 2 | 0.13 | ND |  | ND | ND |  | ND | ND |  | ND | ND |
| 56 | 3 | Exon28 | c.3831C>T | r.3830_3870del41 | p.Asn1278fs | III | c.3870 N (Donor Site) | N | 0.995042 | 0.960065 | 0.034977 | 0.05 | 79.64 | 79.64 | 0 | 7.81 | 7.81 | 0.00 | 0.92 | 0.92 | 0.00 | ND |  | ND | ND |  | ND | 0.02686 |
| 56 | 3 | Exon28 | c.3831C>T | r.3830_3870del41 | p.Asn1278fs | III | c.3829 (**Cryptic** Donor Site) |  | 0 | 0.920268 | 0.920268 | 0.67 | 76.46 | 78.92 | 0.0246 | 0 | 8.1 | 0.51 | 0 | 0.73 | 0.73 | 0 | 3.23 | 0.13 | 59.06 | 86.2 | 0.2714 | ND |
| 57 | 1 | Intron28 | c.3871-13T>A | r.3871_3974del | p.Tyr1292fs | I (3'ss) | c.3871 N (Acceptor Site) | N | 0.999853 | 0.999853 | 0 | 0.65 | 80.65 | 0 | 0.8065 | 8.42 | 2.14 | 0.39 | 0.99 | 0.97 | 0.02 | 5.45 | 0 | 0.26 | 82 | 82 | 0 | 1 |
| 58 | 0 | Intron28 | c. 3871-9_3872delinsG | r.3871_3974del | p.Tyr1292fs | I (3'ss) | c.3871 N (Acceptor Site) | Y | ND |  | ND | 0.98 | 80.65 | 0 | 0.8065 | 8.42 | 0 | 0.53 | 0.99 | 0 | 0.99 | 5.45 | 0 | 0.26 | 82.46 | 0 | 0.8246 | 1 |
| 59 | 3 | Exon29 | c.3974G>A | r.3871_3974del104 | p.Tyr1292fs | I (5'ss) | c.3974 N (Donor Site) | Y | 0.873664 | 0.264975 | 0.608689 | 0.95 | 79.95 | 67.81 | 0.1214 | 7.44 | 2.06 | 0.34 | 0.75 | 0.08 | 0.67 | ND |  | ND | 86.61 | 76.53 | 0.1008 | 0.99932 |
| 60 | 3 | Intron29 | c.3974+1G>T | r.3871_3974del104 | p.Tyr1292fs | I (5'ss) | c.3974 N (Donor Site) | Y | 0.873664 | 0 | 0.873664 | 0.97 | 79.95 | 0 | 0.7995 | 7.44 | 0 | 0.47 | 0.75 | 0 | 0.75 | ND |  | ND | 86.61 | 59.47 | 0.2714 | 1 |
| 61 | 0 | Intron29 | c.3975-2A>T | r.3975_3979del | p.Arg1325fs | IV (3'ss) | c.3975 N (Acceptor Site) | Y | 0.999942 | 0 | 0.999942 | 0.99 | 92.98 | 0 | 0.9298 | 10.82 | 0 | 0.68 | 1 | 0 | 1.00 | 8.34 | 0 | 0.40 | 91.27 | 63.4 | 0.2787 | 1 |
| 61 | 0 | Intron29 | c.3975-2A>T | r.3975_3979del | p.Arg1325fs | IV (3'ss) | c.3980 (**Cryptic** Acceptor Site) | Y | 0.521753 | 0.996559 | 0.474806 | 0.86 | 0 | 82.44 | 0.8244 | 0.67 | 4.85 | 0.26 | 0.17 | 0.69 | 0.52 | 0 | 5.57 | 0.27 | 77 | 77 | 0 | ND |
| 62 | 0 | Intron30 | c.4110+1G>A | r.3975_4110del | p.Arg1325_Gln1370del | I (5'ss) | c.4110 N (Donor Site) | Y | 0.999917 | 0 | 0.999917 | 1.00 | 84.05 | 0 | 0.8405 | 9.37 | 0 | 0.59 | 0.98 | 0 | 0.98 | ND |  | ND | 89.88 | 62.74 | 0.2714 | 1 |
| 63 | 3 | Exon32 | c.4331A>G | r.4174_4332del159 | p.Val1392_Lys1444del | I (5'ss) | c.4332 N (Donor Site) | Y | 0.850215 | 0.162393 | 0.687822 | 0.41 | 83.18 | 74.22 | 0.0896 | 6.38 | 2.65 | 0.23 | 0.41 | 0.16 | 0.25 | ND |  | ND | 87 | 87 | 0 | 0.98874 |
| 64 | 0 | Intron32 | c.4333-2A>G | r.4333_4341del9 | p.Ile1445_Gln1447del | IV (3'ss) | c.4333 N (Acceptor Site) | Y | 0.999931 | 0 | 0.999931 | 0.99 | 83.59 | 0 | 0.8359 | 7.92 | 0 | 0.50 | 0.91 | 0 | 0.91 | 5.47 | 0 | 0.26 | 85.7 | 57.83 | 0.2787 | 1 |
| 64 | 0 | Intron32 | c.4333-2A>G | r.4333_4341del9 | p.Ile1445_Gln1447del | IV (3'ss) | c.4342 (**Cryptic** Acceptor Site) | Y | 0.437561 | 0.998322 | 0.560761 | 0.97 | 0 | 75.64 | 0.7564 | 0 | 3.45 | 0.22 | 0.07 | 0.16 | 0.09 | ND |  | ND | 82.21 | 82.47 | 0.0026 | ND |
| 65 | 1 | Intron33 | c.4431-9T>G | r.4430_4431ins4431-8_4431-1 | p.Arg1477fs | III | c.4431 N (Acceptor Site) | Y | 0.995309 | 0.051267 | 0.944042 | 0.77 | 84.14 | 0 | 0.8414 | 7.3 | 1.28 | 0.38 | 0.79 | 0.47 | 0.32 | 1.38 | 0 | 0.07 | 81 | 81 | 0 | 1 |
| 65 | 1 | Intron33 | c.4431-9T>G | r.4430_4431ins4431-8_4431-1 | p.Arg1477fs | III | c.4431-8 (**Cryptic** Acceptor Site) | Y | 0 | 0.271776 | 0.271776 | 0.60 | 0 | 78.83 | 0.7883 | 0 | 4.28 | 0.27 | 0 | 0.28 | 0.28 | ND |  | ND | 55.6 | 83.47 | -0.2787 | ND |
| 66 | 3 | Intron33 | c.4431-2A>C | r.4431_4447del17 | p.Arg1477fs | IV (3'ss) | c.4431 N (Acceptor Site) | Y | 0.995309 | 0 | 0.995309 | 0.95 | 84.14 | 0 | 0.8414 | 7.3 | 0 | 0.46 | 0.79 | 0 | 0.79 | 1.38 | 0 | 0.07 | 81.5 | 53.63 | 0.2787 | 1 |
| 66 | 3 | Intron33 | c.4431-2A>C | r.4431_4447del17 | p.Arg1477fs | IV (3'ss) | c.4448 (**Cryptic** Acceptor Site) |  | 0.791882 | 0.998992 | 0.20711 | 0.76 | 80.07 | 80.07 | 0 | 4.94 | 6.84 | 0.12 | ND |  | ND | ND |  | ND | 4.94 | 6.84 | -0.019 | ND |
| 67 | 1 | Intron34 | c.4578-21T>C | r.[4577_4578ins4578-14_4578-1, 4577_4578ins4578-17_4578-1] | p.[Arg1526fs, Arg1526fs] | IV | c.4578 N (Acceptor Site) | N | 0.425036 | 0.121527 | 0.303509 | 0.41 | 83.4 | 83.4 | 0 | 2.55 | 2.55 | 0.00 | 0.49 | 0.71 | 0.22 | ND |  | ND | 80.32 | 71.29 | 0.0903 | 0.02686 |
| 67 | 1 | Intron34 | c.4578-21T>C | r.[4577_4578ins4578-14_4578-1, 4577_4578ins4578-17_4578-1] | p.[Arg1526fs, Arg1526fs] | IV | c.4578-14 (**Cryptic** Acceptor Site) | N | 0.470543 | 0.567159 | 0.096616 | 0.14 | ND |  | ND | 3.8 | 3.85 | 0.00 | 0.92 | 0.89 | 0.03 | ND |  | ND | 82 | 82 | 0 | ND |
| 67 | 1 | Intron34 | c.4578-21T>C | r.[4577_4578ins4578-14_4578-1, 4577_4578ins4578-17_4578-1] | p.[Arg1526fs, Arg1526fs] | IV | c.4578-17 (Cryptic Acceptor Site) | N | 0.088561 | 0.198317 | 0.109756 | 0.14 | 82.08 | 82.08 | 0 | 3.12 | 3.03 | 0.01 | 0.39 | 0.27 | 0.12 | ND |  | ND | 86 | 86 | 0 | ND |
| 68 | 1 | Intron34 | c.4578-19A>G | r.4577_4578ins4578-18_4578-1 | p.Asp1527* | III | c.4578 N (Acceptor Site) | N | 0.425036 | 0.096126 | 0.32891 | 0.65 | 83.4 | 83.4 | 0 | 2.55 | 2.83 | 0.02 | 0.49 | 0.49 | 0.00 | ND |  | ND | 80.32 | 50.69 | 0.2963 | 0.01097 |
| 68 | 1 | Intron34 | c.4578-19A>G | r.4577_4578ins4578-18_4578-1 | p.Asp1527* | III | c.4578-18 (**Cryptic** Acceptor Site) | N | 0 | 0.972291 | 0.972291 | 0.99 | 0 | 92.83 | 0.9283 | 0 | 10.22 | 0.64 | 0 | 0.99 | 0.99 | 0 | 6.46 | 0.31 | 67.82 | 95.69 | 0.2787 | ND |
| 69 | 1 | Intron34 | c.4578-3delT | r.[4577_4578ins4578-14_4578-1, 4577_4578ins4578-17_4578-1] | p.[Arg1526fs, Arg1526fs] | IV | c.4578 N (Acceptor Site) | Y | 0.425036 | 0 | 0.425036 | 0.57 | 83.4 | 84.86 | 0.0146 | 2.55 | 2.28 | 0.02 | 0.49 | 0.38 | 0.11 | ND |  | ND | 80 | 80 | 0 | 0.02744 |
| 69 | 1 | Intron34 | c.4578-3delT | r.[4577_4578ins4578-14_4578-1, 4577_4578ins4578-17_4578-1] | p.[Arg1526fs, Arg1526fs] | IV | c.4578-14 (**Cryptic** Acceptor Site) | Y | 0.470543 | 0.756056 | 0.285513 | 0.04 | ND |  | ND | 3.8 | 3.8 | 0.00 | 0.92 | 0.61 | 0.31 | 0 | 2.12 | 0.10 | 82 | 82 | 0 | ND |
| 69 | 1 | Intron34 | c.4578-3delT | r.[4577_4578ins4578-14_4578-1, 4577_4578ins4578-17_4578-1] | p.[Arg1526fs, Arg1526fs] | IV | c.4578-17 (Cryptic Acceptor Site) | Y | 0.088561 | 0.399602 | 0.311041 | 0.04 | 82.08 | 82.08 | 0 | 3.12 | 3.12 | 0.00 | 0.39 | 0.59 | 0.20 | ND |  | ND | 86 | 86 | 0 | ND |
| 70 | 1 | Intron34 | c.4578-3T>G | r.[4577_4578ins4578-14_4578-1, 4577_4578ins4578-17_4578-1] | p.[Arg1526fs, Arg1526fs] | IV | c.4578 N (Acceptor Site) | Y | 0.425036 | 0.007133 | 0.417903 | 0.65 | 83.4 | 78.76 | 0.0464 | 2.55 | 0 | 0.16 | 0.49 | 0 | 0.49 | ND |  | ND | 80 | 80 | 0 | 1 |
| 70 | 1 | Intron34 | c.4578-3T>G | r.[4577_4578ins4578-14_4578-1, 4577_4578ins4578-17_4578-1] | p.[Arg1526fs, Arg1526fs] | IV | c.4578-14 (**Cryptic** Acceptor Site) | Y | 0.470543 | 0.779244 | 0.308701 | 0.40 | ND |  | ND | 3.8 | 3.8 | 0.00 | 0.92 | 0.92 | 0.00 | ND |  | ND | 82 | 82 | 0 | ND |
| 70 | 1 | Intron34 | c.4578-3T>G | r.[4577_4578ins4578-14_4578-1, 4577_4578ins4578-17_4578-1] | p.[Arg1526fs, Arg1526fs] | IV | c.4578-17 (Cryptic Acceptor Site) | Y | 0.088561 | 0.482645 | 0.394084 | 0.40 | 82.08 | 82.08 | 0 | 3.12 | 3.12 | 0.00 | 0.39 | 0.48 | 0.09 | ND |  | ND | 86 | 86 | 0 | ND |
| 71 | 0 | Intron36 | c.4835+2T>G | r.4767_4835del69 | p.Leu1590_Arg1612del | IV (5'ss) | c.4835 N (Donor Site) | Y | 0.998886 | 0 | 0.998886 | 0.99 | 91.62 | 0 | 0.9162 | 10.06 | 0 | 0.63 | 0.99 | 0 | 0.99 | ND |  | ND | 96.21 | 69.07 | 0.2714 | 1 |
| 71 | 0 | Intron36 | c.4835+2T>G | r.4767_4835del69 | p.Leu1590_Arg1612del | IV (5'ss) | c.4766 (**Cryptic** Donor Site) | Y | 0.998886 | 0 | 0.998886 | ND | 59.34 | 59.34 | 0 | ND |  | ND | 0 | 0 | 0 | ND |  | ND | 68.91 | 68.91 | 0 | ND |
| 72 | 0, 3 | Intron36 | c.4836-2A>C | r.4836_5128del293 | p.Phe1613fs | IV (3'ss) | c.4836 N (Acceptor Site) | Y | 0.987256 | 0 | 0.987256 | 0.99 | 83.17 | 0 | 0.8317 | 11.72 | 0 | 0.73 | 1 | 0 | 1.00 | 10.03 | 0 | 0.48 | 84.6 | 56.73 | 0.2787 | 1 |
| 72 | 0, 3 | Intron36 | c.4836-2A>C | r.4836_5128del293 | p.Phe1613fs | IV (3'ss) | c.5128 (**Cryptic** Acceptor Site) | Y | 0.578325 | 0.578325 | 0 | ND | ND |  | ND | ND |  | ND | ND |  | ND | ND |  | ND | ND |  | ND | ND |
| 73 | 1 | Intron37 | c.5269-19C>A | r.5268_5269ins5269-17_5269-1 | p.Val1757fs | III | c.5269 N (Acceptor Site) | N | 0.940376 | 0.620519 | 0.319857 | 0.18 | 83.54 | 83.54 | 0 | 5.95 | 4.04 | 0.12 | 0.81 | 0.73 | 0.08 | 3.63 | 2.36 | 0.06 | 85 | 85 | 0 | 0.27796 |
| 73 | 1 | Intron37 | c.5269-19C>A | r.5268_5269ins5269-17_5269-1 | p.Val1757fs | III | c.5269-17 (**Cryptic** Acceptor Site) | N | 0 | 0.390426 | 0.390426 | 0.76 | 0 | 76.99 | 0.7699 | 0 | 4.79 | 0.30 | 0 | 0.29 | 0.29 | 0 | 1.5 | 0.07 | 50.8 | 78.67 | 0.2787 | ND |
| 74 | 1 | Intron37 | c.5269-14C>G | r.5268_5269ins5269-13_5269-1 | p.Val1757fs | III | c.5269 N (Acceptor Site) | N | 0.940376 | 0.075903 | 0.864473 | 0.87 | 83.54 | 81.13 | 0.0241 | 5.95 | 3.47 | 0.16 | 0.81 | 0.69 | 0.12 | 3.63 | 0 | 0.17 | 85 | 85 | 0 | 0.71743 |
| 74 | 1 | Intron37 | c.5269-14C>G | r.5268_5269ins5269-13_5269-1 | p.Val1757fs | III | c.5269-13 (**Cryptic** Acceptor Site) | N | 0 | 0.723742 | 0.723742 | 0.86 | 0 | 76.98 | 0.7698 | 0 | 3.81 | 0.24 | 0 | 0.32 | 0.32 | 0 | 1.47 | 0.07 | 50.59 | 78.46 | 0.2787 | ND |
| 75 | 1 | Intron37 | c.5269-8C>G | r.5268_5269ins5269-7_5269-1 | p.Val1757fs | III | c.5269 N (Acceptor Site) | Y | 0.940376 | 0.072878 | 0.867498 | 0.88 | 83.54 | 0 | 0.8354 | 5.95 | 0 | 0.37 | 0.81 | 0.53 | 0.28 | 3.63 | 0 | 0.17 | 85 | 85 | 0 | 1 |
| 75 | 1 | Intron37 | c.5269-8C>G | r.5268_5269ins5269-7_5269-1 | p.Val1757fs | III | c.5269-7 (**Cryptic** Acceptor Site) | Y | 0 | 0.827313 | 0.827313 | 0.97 | 0 | 70.38 | 0.7038 | 0 | 4.77 | 0.30 | 0 | 0.03 | 0.03 | 0 | 1.14 | 0.05 | 49.76 | 77.63 | 0.2787 | ND |
| 76 | 0 | Exon38 | c.5609G>T | r.5269_5609del | p.Val1757_Arg1870del | I (5'ss) | c.5609 N (Donor Site) | Y | 0.983083 | 0.531118 | 0.451965 | 0.43 | 80.7 | 68.08 | 0.1262 | 10.15 | 4.15 | 0.38 | 0.97 | 0.06 | 0.91 | 1.86 | 0 | 0.08 | 85.95 | 75.09 | 0.1086 | 0.99828 |
| 77 | 0 | Intron38 | c.5609+1G>T | r.5269_5609del341; r.5269_5812del544 | p.Val1757_Arg1870del;p.Val1757_Ser1938del | I (5'ss) | c.5609 N (Donor Site) | Y | 0.983083 | 0 | 0.983083 | 0.99 | 80.7 | 0 | 0.807 | 10.15 | 0 | 0.63 | 0.97 | 0 | 0.97 | 1.86 | 0 | 0.08 | 86.49 | 59.35 | 0.2714 | 1 |
| 78 | 3 | Intron39 | c.5812+332A>G | r.5812_5813ins5812+155_5812+331 | premature stop codon | II | c.5812+331 (**Cryptic** Donor Site) | N | 0 | 0.973091 | 0.973091 | 0.75 | 0 | 86.69 | 0.8669 | 0 | 10.16 | 0.64 | 0 | 1 | 1 | 0 | 2.39 | 0.10 | 61.7 | 88.85 | -0.2715 | 0.02686 |
| 78 | 3 | Intron39 | c.5812+332A>G | r.5812_5813ins5812+155_5812+331 | premature stop codon | II | c.5812+155 (**Cryptic** Acceptor Site) | N | 0.104477 | 0.624831 | 0.520354 | 0.44 | 75.57 | 75.57 | 0 | 1.24 | 1.24 | 0.00 | 0 | 0 | 0 | ND |  | ND | ND |  | ND | ND |
| 79 | 1 | Intron39 | c.5813-12_5813-9delATTT | r.5812_5813ins5813-11_5813-1 (of mutated allele = r.5812_5813ins TGTTTTTCCAG) | p.Ser1938fs | IV | c.5813 N (Acceptor Site) | Y | 0.998032 | 0 | 0.998032 | 0.25 | 98.76 | 0 | 0.9876 | 8.44 | 4.14 | 0.27 | 0.98 | 0.82 | 0.16 | 6.5 | 1.58 | 0.23 | ND |  | ND | 1 |
| 79 | 1 | Intron39 | c.5813-12_5813-9delATTT | r.5812_5813ins5813-11_5813-1 (of mutated allele = r.5812_5813ins TGTTTTTCCAG) | p.Ser1938fs | IV | c.5813-11 (**Cryptic** Acceptor Site) | Y | 0.998032 | 0 | 0.998032 | 0.31 | ND |  | ND | ND |  | ND | ND |  | ND | ND |  | ND | ND |  | ND | ND |
| 80 | 0 | Exon40 | c.6006G>C | r.6003_6006delGCAG | p.Gln2002fs | IV (5'ss) ? | c.6006 N (Donor Site) | Y | 0.887336 | 0.16634 | 0.720996 | 0.94 | 72.41 | 59.1 | 0.1331 | 8.35 | 0 | 0.52 | 0.58 | 0 | 0.58 | ND |  | ND | 80.51 | 69.35 | 0.1116 | 0.99999 |
| 80 | 0 | Exon40 | c.6006G>C | r.6003_6006delGCAG | p.Gln2002fs | IV (5'ss) ? | c.6002 (**Cryptic** Donor Site) | Y | 0 | 0 | 0 | ND | 74.07 | 73.37 | 0.007 | ND |  | ND | ND |  | ND | ND |  | ND | ND |  | ND | ND |
| 81 | 3 | Exon40 | c.6006G>A | r.6003_6006del4 | p.Gln2002fs | IV (5'ss) | c.6006 N (Donor Site) | Y | 0.887336 | 0.205349 | 0.681987 | 0.94 | 72.41 | 60.28 | 0.1213 | 8.35 | 0 | 0.52 | 0.58 | 0 | 0.58 | ND |  | ND | 80.51 | 70.42 | 0.1009 | 0.99998 |
| 81 | 3 | Exon40 | c.6006G>A | r.6003_6006del4 | p.Gln2002fs | IV (5'ss) | c.6002 (**Cryptic** Donor Site) |  | 0 | 0 | 0 | 0.84 | 74.07 | 83.82 | 0.0975 | ND |  | ND | ND |  | ND | ND |  | ND | ND |  | ND | ND |
| 82 | 1 | Intron40 | c.6007-5A>G | r.6006_6007ins6007-4_6007-1 | p.Ile2003fs | III | c.6007 N (Acceptor Site) | Y | 0.999218 | 0.983552 | 0.015666 | 0.06 | 77.21 | 0 | 0.7721 | 8.12 | 3.38 | 0.30 | 0.78 | 0.74 | 0.04 | 6.65 | 0 | 0.32 | 81 | 81 | 0 | 1 |
| 82 | 1 | Intron40 | c.6007-5A>G | r.6006_6007ins6007-4_6007-1 | p.Ile2003fs | III | c.6007-4 (**Cryptic** Acceptor Site) | Y | 0 | 0.997967 | 0.997967 | 1.00 | 0 | 90.77 | 0.9077 | 0 | 9.33 | 0.58 | 0 | 0.98 | 0.98 | 0 | 4.55 | 0.22 | 63.45 | 91.32 | 0.2787 | ND |
| 83 | 1 | Intron41 | c.6148-16T>G | r.6148_6427del | p.Val2050fs | I (3'ss) | c.6148 N (Acceptor Site) | N | 0.987466 | 0.931079 | 0.056387 | 0.42 | 93.19 | 93.19 | 0 | 8.65 | 7.21 | 0.09 | 0.99 | 0.98 | 0.01 | 5.33 | 1.91 | 0.16 | 91 | 91 | 0 | 0.09734 |
| 84 | 1 | Intron41 | c.6148-13T>A | r.6148_6427del | p.Val2050fs | I (3'ss) | c.6148 N (Acceptor Site) | N | 0.987466 | 0.701769 | 0.285697 | 0.74 | 93.19 | 0 | 0.9319 | 8.65 | 3.25 | 0.34 | 0.99 | 0.97 | 0.02 | 5.33 | 0 | 0.25 | 91 | 91 | 0 | 1 |
| 85 | 1 | Intron41 | c.6148-3C>A | r.6148_6427del | p.Val2050fs | I (3'ss) | c.6148 N (Acceptor Site) | Y | 0.987466 | 0.844095 | 0.143371 | 0.77 | 93.19 | 83.22 | 0.0997 | 8.65 | 7.03 | 0.10 | 0.99 | 0.93 | 0.06 | 5.33 | 3.44 | 0.09 | 91 | 91 | 0 | 0.83134 |
| 86 | 0 | Intron42 | c.6427+1G>T | r.6148_6427del | p.Val2050fs | I (5'ss) | c.6427 N (Donor Site) | Y | 0.854717 | 0 | 0.854717 | 0.82 | 88.43 | 0 | 0.8843 | 10.36 | 0 | 0.65 | 1 | 0 | 1.00 | 1.03 | 0 | 0.04 | 93.13 | 65.99 | 0.2714 | 1 |
| 87 | 1 | Intron42 | c.6428-11T>G | r.6428_6642del | p.Glu2143fs | I (3'ss) | c. 6428 N (Acceptor Site) | Y | 0.9812 | 0.072435 | 0.908765 | 0.89 | 93.2 | 0 | 0.932 | 9.6 | 4.48 | 0.32 | 0.93 | 0.7 | 0.23 | 7.46 | 2.25 | 0.25 | 90 | 90 | 0 | 1 |
| 88 | 1, 3 | Intron42 | c.6428-3C>A | r.6428_6642del | p.Glu2143fs | I (3'ss) | c. 6428 N (Acceptor Site) | Y | 0.979464 | 0.464666 | 0.514798 | 0.11 | 93.2 | 83.24 | 0.0996 | 9.6 | 5.15 | 0.28 | 0.93 | 0.41 | 0.52 | 7.46 | 4.88 | 0.12 | 90 | 90 | 0 | 0.98019 |
| 89 | 1 | Intron42 | c.6428-3C>G | r.6427_6428ins6428-2_6428-1 | p.Glu2143fs | III | c.6428 N (Acceptor Site) | Y | 0.9812 | 0.013829 | 0.967371 | 0.74 | 93.2 | 82.38 | 0.1082 | 9.6 | 0.14 | 0.59 | 0.93 | 0.02 | 0.91 | 7.46 | 0 | 0.36 | 90 | 90 | 0 | 0.99978 |
| 89 | 1 | Intron42 | c.6428-3C>G | r.6427_6428ins6428-2_6428-1 | p.Glu2143fs | III | c.6428-2 (**Cryptic** Acceptor Site) | Y | 0 | 0.013829 | 0.013829 | 0.91 | 0 | 83.62 | 0.8362 | 0 | 7.64 | 0.48 | 0 | 0.98 | 0.98 | 0 | 4.26 | 0.20 | 56.94 | 84.81 | 0.2787 | ND |
| 90 | 1 | Intron42 | c.6428-3delC | r.[6428_6642del, 6428_6542del] | p.[Glu2143fs, Glu2143fs] | I (3'ss), IV | c.6428 N (Acceptor Site) | Y | 0.979464 | 0 | 0.979464 | 0.24 | 93.2 | 82.63 | 0.1057 | 9.6 | 4.56 | 0.32 | 0.93 | 0.8 | 0.13 | 7.46 | 4.34 | 0.15 | 90 | 90 | 0 | 0.9879 |
| 90 | 1 | Intron42 | c.6428-3delC | r.[6428_6642del, 6428_6542del] | p.[Glu2143fs, Glu2143fs] | I (3'ss), IV | c.6543 (**Cryptic** Acceptor Site) | Y | 0.979464 | 0 | 0.979464 | ND | 82.5 | 0 | 0.825 | 8.63 | 0 | 0.54 | 0.82 | 0 | 0.82 | 4.2 | 0 | 0.20 | ND |  | ND | ND |
| 91 | 0 | Intron44 | c.6704+1G>A | r.6643_6704del62 | p.Ala2215_Arg2235del | I (5'ss) | c.6704 N (Donor Site) | Y | 0.999293 | 0 | 0.999293 | 1.00 | 89.52 | 0 | 0.8952 | 9.79 | 0 | 0.61 | 1 | 0 | 1.00 | 3.49 | 0 | 0.15 | 92.66 | 65.52 | 0.2714 | 1 |
| 92 | 1 | Intron44 | c.6705-17G>A | r.6705_6819del | p.Phe2236fs | I (3'ss) | c.6705 N (Acceptor Site) | N | 0.988564 | 0.865145 | 0.123419 | 0.75 | 92.97 | 92.97 | 0 | 7.85 | 6.69 | 0.07 | 0.56 | 0.6 | 0.04 | 2.28 | 0 | 0.11 | 91 | 91 | 0 | 0.08447 |
| 93 | 1 | Intron44 | c.6705-7_6705-6insAAAA | r.6705_6819del | p.Phe2236fs | I (3'ss) | c.6705 N (Acceptor Site) | Y | 0.988564 | 0.919577 | 0.068987 | 0.71 | 92.97 | 71.74 | 0.2123 | 7.85 | 6.54 | 0.08 | 0.56 | 0 | 0.56 | 2.28 | 0 | 0.11 | ND |  | ND | 0.02686 |
| 94 | 1 | Intron44 | c.6705-5T>G | r.6705_6819del | p.Phe2236fs | I (3'ss) | c.6705 N (Acceptor Site) | Y | 0.988564 | 0.780088 | 0.208476 | 0.73 | 92.97 | 86.56 | 0.0641 | 7.85 | 6 | 0.12 | 0.56 | 0.04 | 0.52 | 2.28 | 0 | 0.11 | 91 | 91 | 0 | 0.64021 |
| 95 | 1 | Intron44 | c.6705-6T>G | r.6705_6819del | p.Phe2236fs | I (3'ss) | c.6705 N (Acceptor Site) | Y | 0.988564 | 0.088346 | 0.900218 | 0.82 | 92.97 | 0 | 0.9297 | 7.85 | 1.82 | 0.38 | 0.56 | 0.05 | 0.51 | 2.28 | 0 | 0.11 | 91 | 91 | 0 | 1 |
| 96 | 1 | Intron44 | c.6705-3C>A | r.6705_6819del | p.Phe2236fs | I (3'ss) | c.6705 N (Acceptor Site) | Y | 0.988564 | 0.063174 | 0.92539 | 0.82 | 92.97 | 83.01 | 0.0996 | 7.85 | 4.22 | 0.23 | 0.56 | 0.01 | 0.55 | 2.28 | 0 | 0.11 | 91 | 91 | 0 | 0.97987 |
| 97 | 1 | Intron44 | c.6705-3C>G | r.6705_6819del | p.Phe2236fs | I (3'ss) | c.6705 N (Acceptor Site) | Y | 0.988564 | 0.013972 | 0.974592 | 0.82 | 92.97 | 82.15 | 0.1082 | 7.85 | 0 | 0.49 | 0.56 | 0 | 0.56 | 2.28 | 0 | 0.11 | 91 | 91 | 0 | 0.99983 |
| 98 | 0 | Exon45 | c.6733C>T | r.6705_6819del115 | p.Phe2236fs | V | c.6705 N (Acceptor Site) | N | 0.988564 | 0.951458 | 0.037106 | 0.34 | 92.97 | 92.97 | 0 | 7.85 | 7.85 | 0.00 | 0.56 | 0.56 | 0.00 | 2.28 | 2.1 | 0.01 | 90.42 | 90.42 | 0 | 0.02686 |
| 99 | 1 | Intron45 | c.6820-10T>G | r.6820_6921del | p.Ala2274_Lys2307del | I (3'ss) | c.6820 N (Acceptor Site) | Y | 0.965303 | 0.811142 | 0.154161 | 0.12 | 84.71 | 78.86 | 0.0585 | 6.04 | 5.39 | 0.04 | 0.09 | 0.02 | 0.07 | 3.44 | 2.25 | 0.06 | 84 | 84 | 0 | 0.3516 |
| 100 | 1 | Intron45 | c.6820-9T>G | r.6820_6921del | p.Ala2274_Lys2307del | I (3'ss) | c.6820 N (Acceptor Site) | Y | 0.965303 | 0.839509 | 0.125794 | 0.11 | 84.71 | 79.28 | 0.0543 | 6.04 | 4.8 | 0.08 | 0.09 | 0.01 | 0.08 | 3.44 | 0 | 0.16 | 84 | 84 | 0 | 0.52594 |
| 101 | 0 | Intron46 | c.6922-3C>G | r.? | premature stop codon | I (3'ss) | c.6922 N (Acceptor Site) | Y | 0.984198 | 0.165303 | 0.818895 | 0.74 | 92.26 | 81.44 | 0.1082 | 6.23 | 1.16 | 0.32 | 0.93 | 0.01 | 0.92 | 1.45 | 0 | 0.07 | 91.48 | 81.18 | 0.103 | 0.9991 |
| 102 | 3 | Intron47 | c.7062+1G>A | r.[6922_7062del141,  6820_7062del243,  6705_7062del358] | p.Asp2308_Lys2354del, Ala2274_lys2354del, 2236fs | I (5'ss) | c.7062 N (Donor Site) | Y | 0.994958 | 0 | 0.994958 | 0.96 | 93.91 | 0 | 0.9391 | 10.22 | 0 | 0.64 | 1 | 0 | 1.00 | 2.78 | 0 | 0.12 | 96.93 | 69.79 | 0.2714 | 1 |
| 103 | 1 | Intron47 | c.7063-10T>G | r.7063_7189del, r.7062_7063ins7063-9_7063-1 | p.Ser2355fs, p.Lys2354_Ser2355insLeu* | I (3'ss), III | c.7063 N (Acceptor Site) | Y | 0.996004 | 0.029438 | 0.966566 | 0.39 | 79.1 | 0 | 0.791 | 4.48 | 0 | 0.28 | 0.47 | 0.12 | 0.35 | 1.49 | 0 | 0.07 | 81 | 81 | 0 | 1 |
| 103 | 1 | Intron47 | c.7063-10T>G | r.7063_7189del, r.7062_7063ins7063-9_7063-1 | p.Ser2355fs, p.Lys2354_Ser2355insLeu* | I (3'ss), III | c.7063-9 (**Cryptic** Acceptor Site) | Y | 0.996004 | 0.029438 | 0.966566 | 0.70 | 0 | 65.47 | 65.47 | 0 | 2.64 | 0.17 | 0 | 0.01 | 0.01 | ND |  | ND | 48.28 | 76.15 | -0.2787 | ND |
| 104 | 1, 3 | Intron47 | c.7063-4A>G | r.7062_7063ins7063-3_7063-1 | p.Ser2355* | IV (3'ss) | c.7063 N (Acceptor Site) | Y | 0.996004 | 0.901589 | 0.094415 | 0.03 | 79.1 | 0 | 0.791 | 4.48 | 1.52 | 0.19 | 0.47 | 0.27 | 0.20 | 1.49 | 0 | 0.07 | 81 | 81 | 0 | 0.86354 |
| 104 | 1, 3 | Intron47 | c.7063-4A>G | r.7062_7063ins7063-3_7063-1 | p.Ser2355* | IV (3'ss) | c.7063-3 (**Cryptic** Acceptor Site) | Y | 0 | 0.903852 | 0.903852 | 0.98 | 0 | 72.7 | 0.727 | 0 | 2.47 | 0.15 | 0 | 0.37 | 0.37 | ND |  | ND | 50.57 | 78.44 | -0.2787 | ND |
| 105 | 1 | Intron47 | c.7063-3T>G | r.7062_7063ins7063-2_7063-1 | p.Ser2355fs | III | c.7063 N (Acceptor Site) | Y | 0.996004 | 0.060889 | 0.935115 | 0.54 | 79.1 | 74.46 | 0.0464 | 4.48 | 0 | 0.28 | 0.47 | 0 | 0.47 | 1.49 | 0 | 0.07 | 81 | 81 | 0 | 1 |
| 105 | 1 | Intron47 | c.7063-3T>G | r.7062_7063ins7063-2_7063-1 | p.Ser2355fs | III | c.7063-2 (**Cryptic** Acceptor Site) | Y | 0 | 0.892868 | 0.892868 | 0.89 | 0 | 77.64 | 0.7764 | 0 | 3.87 | 0.24 | 0 | 0.17 | 0.17 | ND |  | ND | 53.29 | 81.16 | -0.2787 | ND |
| 106 | 1 | Intron48 | c.7190-20T>A | r.7189_7190ins7190-18_7190-1, r.7190_7321del | p.Lys2396_Gly2397ValCysLeuPhePheVal, p.Gly2397_Ala2440del | III | c.7190 N (Acceptor Site) | N | 0.999697 | 0.98849 | 0.011207 | 0.81 | 92.54 | 92.54 | 0 | 10.11 | 8.29 | 0.11 | 1 | 1 | 0.00 | 8.73 | 6.38 | 0.11 | 89 | 89 | 0 | 0.10792 |
| 106 | 1 | Intron48 | c.7190-20T>A | r.7189_7190ins7190-18_7190-1, r.7190_7321del | p.Lys2396_Gly2397ValCysLeuPhePheVal, p.Gly2397_Ala2440del | III | c.7190-18 (**Cryptic** Acceptor Site) | N | 0.999697 | 0.98849 | 0.011207 | 0.05 | 0 | 80.14 | 0.8014 | 0 | 6.73 | 0.42 | 0 | 0.93 | 0.93 | 0 | 2.01 | 0.10 | 56.32 | 84.19 | 0.2787 | ND |
| 107 | 1 | Intron48 | c.7190-12T>A | r.7189_7190ins7190-10_7190-1, r.7190_7321del | p.Gly2397fs, p.Gly2397_Ala2440del | III | c.7190 N (Acceptor Site) | Y | 0.999697 | 0.711066 | 0.288631 | 0.98 | 92.54 | 0 | 0.9254 | 10.11 | 3.83 | 0.39 | 1 | 0.99 | 0.01 | 8.73 | 0 | 0.42 | 89 | 89 | 0 | 1 |
| 107 | 1 | Intron48 | c.7190-12T>A | r.7189_7190ins7190-10_7190-1, r.7190_7321del | p.Gly2397fs, p.Gly2397_Ala2440del | III | c.7190-10 (**Cryptic** Acceptor Site) | Y | 0 | 0.961433 | 0.961433 | 0.88 | 0 | 80.14 | 0.8014 | 0 | 6.99 | 0.44 | 0 | 0.92 | 0.92 | 0 | 4.89 | 0.23 | 56.32 | 84.19 | 0.2787 | ND |
| 108 | 0 | Intron50 | c.7458-2A>G | r.7458_7615del158 | p.Thr2487fs | I (3'ss) | c.7458 N (Acceptor Site) | Y | 0.998248 | 0 | 0.998248 | 1.00 | 97.12 | 0 | 0.9712 | 11.72 | 0 | 0.73 | 0.98 | 0 | 0.98 | 10.93 | 0 | 0.52 | 92.22 | 64.35 | 0.2787 | 1 |
| 109 | 3 | Intron52 | c.7738+1G>A | r.7738_7739ins7738+1_7738+39,7616_7738del123 | premature stop codon | I (5'ss) | c.7738 N (Donor Site) | Y | 0.982835 | 0 | 0.982835 | 0.98 | 77.22 | 0 | 0.7722 | 6.04 | 0 | 0.38 | 0.77 | 0 | 0.77 | ND |  | ND | 86.88 | 59.71 | 0.2717 | 1 |
| 110 | 1 | Intron52 | c.7739-3C>G | r.[7739_7869del, 7616_7869del] | p.[Glu2580fs, Thr2540fs] | I (3'ss) | c.7739 N (Acceptor Site) | Y | 0.981000 | 0.039833 | 0.941167 | 0.71 | 87.08 | 76.26 | 0.1082 | 7.79 | 0 | 0.49 | 0.91 | 0.01 | 0.90 | 4.75 | 0 | 0.23 | 85 | 85 | 0 | 1 |
| 111 | 1 | Intron53 | c.7870-24_7870-19delinsTTTTAG | r.7870_7970del | p.Thr2625* | I (3'ss) | c.7870 N (Acceptor Site) | N | 0.993741 | 0.863043 | 0.130698 | Error | 85.84 | 85.84 | 0 | 8.18 | 6.27 | 0.12 | 0.52 | 0.37 | 0.15 | 3.06 | 1.17 | 0.09 | ND |  | ND | 0.15858 |
| 112 | 1 | Intron53 | c.7870-3C>G | r.7870_7970del | p.Thr2625* | I (3'ss) | c.7870 N (Acceptor Site) | Y | 0.993741 | 0.468665 | 0.525076 | 0.71 | 85.84 | 75.02 | 0.1082 | 8.18 | 0.9 | 0.46 | 0.52 | 0 | 0.52 | 3.06 | 0 | 0.15 | 81 | 81 | 0 | 0.99962 |
| 113 | 1 | Intron54 | c.7971-17C>G | r.7971_8113del | p.His2658fs | I (3'ss) | c.7971 N (Acceptor Site) | N | 0.997756 | 0.982635 | 0.015121 | 0.33 | 93.82 | 93.82 | 0 | 11.38 | 9.92 | 0.09 | 1 | 1 | 0.00 | 10.33 | 7.73 | 0.12 | 93 | 93 | 0 | 0.07372 |
| 114 | 1 | Intron56 | c.8161-3C>G | r.8160_8161ins8161-2_8161-1 | p.Gln2721fs | III | c.8161 N (Acceptor Site) | Y | 0.994386 | 0.030855 | 0.963531 | 0.97 | 89.42 | 78.6 | 0.1082 | 10.12 | 2.11 | 0.50 | 0.82 | 0 | 0.82 | 5.78 | 0 | 0.28 | 90 | 90 | 0 | 0.99904 |
| 114 | 1 | Intron56 | c.8161-3C>G | r.8160_8161ins8161-2_8161-1 | p.Gln2721fs | III | c.8161-2 (**Cryptic** Acceptor Site) | Y | 0 | 0.914576 | 0.914576 | 0.95 | 0 | 81.27 | 0.8127 | 0 | 6.8 | 0.43 | 0 | 0.92 | 0.92 | 0 | 4.35 | 0.21 | 53.42 | 81.29 | 0.2787 | ND |
| ^a^0: St. Mary's Hospital; 1: Wimmer et al., (2019); 2: Inha University Hospital; 3: Wimmer et al., (2008); ND: Not detected. | | | | | | | | | | | | | | | | | | | | | | | | | | | | |

| Assessed Negative Variant No. | Gene | cDNA variant | Affected Splice Site  (N=natural, Cryptic) | located at 5' and 3' consensus site: (Cartegni et al. 2002) | wt  [SpliceRover] | mut  [SpliceRover] | delta  [SpiceRover] | SpliceAI | wt  [SSF] | mt  [SSF] | delta  [SSF] | wt  [MES] | mt  [MES] | delta  [MES] | wt  [NNSplice] | mt  [NNSplice] | delta  [NNSplice] | wt  [GeneSplicer] | mt  [Gene  Splicer] | delta  [GeneSplicer] | delta  [HSF] | SPiCEprobability |
| --- | --- | --- | --- | --- | --- | --- | --- | --- | --- | --- | --- | --- | --- | --- | --- | --- | --- | --- | --- | --- | --- | --- |
| 1 | BRCA1 | c.81-65G>C | c.81 N (Acceptor Site) | N | 0.990283 | 0.991408 | 0.001125 | 0.00 | 70.09 | 70.09 | 0.7009 | 7.05 | 7.05 | 0.40 | 0.52 | 0.52 | 0.12 | 4.46 | 4.4 | 0.21 | 0 | 0.02686 |
| 2 | BRCA1 | c.81-49A>C | c.81 N (Acceptor Site) | N | 0.990283 | 0.988733 | 0.00155 | 0.01 | 70.09 | 70.09 | 0.7008 | 7.05 | 7.05 | 0.40 | 0.52 | 0.52 | 0.12 | 4.46 | 4.48 | 0.21 | 0 | 0.02686 |
| 3 | BRCA1 | c.81-14C>T | c.81 N (Acceptor Site) | N | 0.990283 | 0.99281 | 0.002527 | 0.00 | 70.09 | 72.87 | 0.7009 | 7.05 | 7.26 | 0.40 | 0.52 | 0.58 | 0.12 | 4.46 | 4.88 | 0.21 | 0 | 0.00786 |
| 4 | BRCA1 | c.81-13C>G | c.81 N (Acceptor Site) | N | 0.990283 | 0.987116 | 0.003167 | 0.00 | 70.09 | 67.61 | 0.7009 | 7.05 | 6.04 | 0.40 | 0.52 | 0.3 | 0.12 | 4.46 | 3.06 | 0.21 | 0 | 0.24528 |
| 5 | BRCA1 | c.81-13C>A | c.81 N (Acceptor Site) | N | 0.990283 | 0.986035 | 0.004248 | 0.01 | 70.09 | 67.4 | 0.7008 | 7.05 | 5.26 | 0.40 | 0.52 | 0.25 | 0.12 | 4.46 | 2.63 | 0.21 | 0 | 0.49529 |
| 6 | BRCA1 | c.442-25G>A | c.442 N (Acceptor Site) | N | 0.984142 | 0.992206 | 0.008064 | 0.01 | 66.72 | 66.72 | 66.71 | 3.71 | 3.71 | 3.94 | 0.08 | 0.08 | 3.8575 | 2.47 | 3.02 | 0.07 | 0 | 0.02686 |
| 7 | BRCA1 | c.442-16T>G | c.442 N (Acceptor Site) | N | 0.984142 | 0.95409 | 0.030052 | 0.06 | 66.72 | 66.72 | 66.66 | 3.71 | 2.76 | 3.93 | 0.08 | 0.02 | 3.854375 | 2.47 | 1.84 | 0.07 | 0 | 0.18419 |
| 8 | BRCA1 | c.548-17G>T | c.548 N (Acceptor Site) | N | 0.988003 | 0.990619 | 0.002616 | 0.00 | 69.11 | 69.11 | 69.11 | 2.82 | 3.76 | 4.14 | 0.01 | 0.03 | 4.133125 | ND |  | ND | 0 | 0.00176 |
| 9 | BRCA1 | c.594-34T>C | c.594 N (Acceptor Site) | N | 0.860468 | 0.856919 | 0.003549 | 0.02 | 83.28 | 83.28 | 0.8326 | 10.00 | 10.00 | 0.57 | 0.98 | 0.98 | 0.41 | 4.51 | 4.49 | 0.20 | 0 | 0.02686 |
| 10 | BRCA1 | c.594-15G>C | c.594 N (Acceptor Site) | N | 0.860468 | 0.904944 | 0.044476 | 0.00 | 83.28 | 83.28 | 0.8328 | 10.00 | 10.2 | 0.57 | 0.98 | 0.99 | 0.41 | 4.51 | 5.1 | 0.20 | 0 | 0.02288 |
| 11 | BRCA1 | c.4097-29C>T | c.4097 N (Acceptor Site) | N | 0.999415 | 0.999373 | 4.2E-05 | 0.00 | 89.19 | 89.19 | 0.8919 | 8.15 | 8.15 | 0.45 | 0.89 | 0.89 | 0.44 | 10.7 | 10.67 | 0.49 | 0 | 0.02686 |
| 12 | BRCA1 | c.5153-34T>G | c.5153 N (Acceptor Site) | N | 0.998952 | 0.996078 | 0.002874 | 0.01 | 81.95 | 81.95 | 0.8194 | 8.78 | 8.78 | 0.50 | 0.96 | 0.96 | 0.46 | 5.11 | 4.7 | 0.22 | 0 | 0.02686 |
| 13 | BRCA1 | c.5153-13A>G | c.5153 N (Acceptor Site) | N | 0.998952 | 0.999273 | 0.000321 | 0.00 | 81.95 | 82.09 | 0.8195 | 8.78 | 10.67 | 0.50 | 0.96 | 0.97 | 0.46 | 5.11 | 5.76 | 0.22 | 0 | 0.00437 |
| 14 | BRCA1 | c.212+23T>A | c.212 N (Donor Site) | N | 0.619023 | 0.616254 | 0.002769 | 0.04 | 77.37 | 77.37 | 0.7733 | 7.84 | 7.84 | 0.44 | 0.92 | 0.92 | 0.48 | 2.81 | 2.65 | 0.10 | 0 | 0.02686 |
| 15 | BRCA1 | c.441+18C>T | c.441 N (Donor Site) | N | 0.9977 | 0.997547 | 0.000153 | 0.00 | 69.88 | 69.88 | 69.88 | 3.23 | 3.23 | 4.17 | 0.39 | 0.39 | 3.775625 | ND |  | ND | 0 | 0.02686 |
| 16 | BRCA1 | c.441+51T>C | c.441 N (Donor Site) | N | 0.9977 | 0.997628 | 7.2E-05 | 0.00 | 69.88 | 69.88 | 69.88 | 3.23 | 3.23 | 4.17 | 0.39 | 0.39 | 3.775625 | ND |  | ND | 0 | 0.02686 |
| 17 | BRCA1 | c.4357+10G>A | c.4357 N (Donor Site) | N | 0.98263 | 0.983081 | 0.000451 | 0.00 | 85.16 | 85.16 | 0.8516 | 6.64 | 6.64 | 0.36 | 0.99 | 0.99 | 0.63 | 5.43 | 4.79 | 0.20 | 0 | 0.02686 |
| 18 | BRCA1 | c.4357+59C>A | c.4357 N (Donor Site) | N | 0.98263 | 0.985033 | 0.002403 | 0.00 | 85.16 | 85.16 | 0.8516 | 6.64 | 6.64 | 0.36 | 0.99 | 0.99 | 0.63 | 5.43 | 5.25 | 0.20 | 0 | 0.02686 |
| 19 | BRCA1 | c.4484+14A>G | c.4484 N (Donor Site) | N | 0.980313 | 0.977231 | 0.003082 | 0.01 | 94.36 | 94.36 | 0.9435 | 10.57 | 10.57 | 0.60 | 1.00 | 1.00 | 0.40 | 2.15 | 2.14 | 0.07 | 0 | 0.02686 |
| 20 | BRCA1 | c.5074+92T>C | c.5074 N (Donor Site) | N | 0.399591 | 0.398879 | 0.000712 | 0.00 | 71.9 | 71.9 | 0.719 | 7.48 | 7.48 | 0.42 | 0.92 | 0.92 | 0.50 | ND |  | ND | 0 | 0.02686 |
| 21 | BRCA1 | c.5152+85delT | c.5152 N (Donor Site) | N | 0.994517 | 0.994173 | 0.000344 | 0.00 | 74.34 | 74.34 | 0.7434 | 7.96 | 7.96 | 0.45 | 0.95 | 0.95 | 0.50 | 2.25 | 2.25 | 0.07 | 0 | 0.02686 |
| 22 | BRCA1 | c.5152+94T>G | c.5152 N (Donor Site) | N | 0.994517 | 0.995552 | 0.001035 | 0.00 | 74.34 | 74.34 | 0.7434 | 7.96 | 7.96 | 0.45 | 0.95 | 0.95 | 0.50 | 2.25 | 2.25 | 0.07 | 0 | 0.02686 |
| 23 | BRCA1 | c.5193+64T>G | c.5193 N (Donor Site) | N | 0.988227 | 0.977481 | 0.010746 | 0.00 | 96.95 | 96.95 | 0.9695 | 11.08 | 11.08 | 0.63 | 1.00 | 1.00 | 0.37 | 3.51 | 3.51 | 0.13 | 0 | 0.02686 |
| 24 | BRCA1 | c.5277+48_59 dup12 | c.5277 N (Donor Site) | N | 0.998575 | 0.998685 | 0.00011 | 0.00 | 82.52 | 82.52 | 0.8252 | 9.06 | 9.06 | 0.51 | 0.93 | 0.93 | 0.42 | 7.25 | 7.25 | 0.28 | 0 | 0.02686 |
| 25 | BRCA1 | c.5277+78G>A | c.5277 N (Donor Site) | N | 0.998575 | 0.998877 | 0.000302 | 0.00 | 82.52 | 82.52 | 0.8252 | 9.06 | 9.06 | 0.51 | 0.93 | 0.93 | 0.42 | 7.25 | 7.37 | 0.28 | 0 | 0.02686 |
| 26 | BRCA1 | c.5332+13G>T | c.5332 N (Donor Site) | N | 0.989235 | 0.991207 | 0.001972 | 0.00 | 94.67 | 94.67 | 0.9467 | 10.77 | 10.77 | 0.61 | 1.00 | 1.00 | 0.39 | 8.13 | 7.48 | 0.32 | 0 | 0.02686 |
| 27 | BRCA1 | c.5332+39C>T | c.5332 N (Donor Site) | N | 0.989235 | 0.988354 | 0.000881 | 0.00 | 94.67 | 94.67 | 0.9467 | 10.77 | 10.77 | 0.61 | 1.00 | 1.00 | 0.39 | 8.13 | 8.01 | 0.32 | 0 | 0.02686 |
| 28 | BRCA1 | c.5406+33A>T | c.5406 N (Donor Site) | N | 0.988564 | 0.994192 | 0.005628 | 0.00 | 78.64 | 78.64 | 0.7864 | 9.49 | 9.49 | 0.54 | 0.98 | 0.98 | 0.44 | 7.86 | 7.73 | 0.31 | 0 | 0.02686 |
| 29 | BRCA1 | c.5406+45C>A | c.5406 N (Donor Site) | N | 0.988564 | 0.981916 | 0.006648 | 0.00 | 78.64 | 78.64 | 0.7864 | 9.49 | 9.49 | 0.54 | 0.98 | 0.98 | 0.44 | 7.86 | 7.75 | 0.31 | 0 | 0.02686 |
| 30 | BRCA2 | c.426-24G>A | c.426 N (Acceptor Site) | N | 0.991665 | 0.993453 | 0.001788 | 0.02 | 86.73 | 86.73 | 0.8671 | 8.99 | 8.99 | 0.51 | 0.99 | 0.99 | 0.48 | ND |  | ND | 0 | 0.02686 |
| 31 | BRCA2 | c.476-24A>G | c.476 N (Acceptor Site) | N | 0.995799 | 0.994868 | 0.000931 | 0.01 | 88.73 | 88.73 | 0.8872 | 10.49 | 10.49 | 0.60 | 0.91 | 0.91 | 0.31 | 2.02 | 0 | 0.08 | 0 | 0.02686 |
| 32 | BRCA2 | c.517-19C>T | c.517 N (Acceptor Site) | N | 0.95829 | 0.949211 | 0.009079 | 0.00 | 94.42 | 94.42 | 0.9442 | 10 | 9.82 | 0.57 | 0.98 | 0.99 | 0.41 | 8.28 | 7.78 | 0.37 | 0 | 0.031 |
| 33 | BRCA2 | c.1910-51G>T | c.1910 N (Acceptor Site) | N | 0.997401 | 0.997612 | 0.000211 | 0.00 | 87.93 | 87.93 | 0.8793 | 10.77 | 10.77 | 0.62 | 0.98 | 0.98 | 0.36 | 6.50 | 6.60 | 0.29 | 0 | 0.02686 |
| 34 | BRCA2 | c.6938-26T>C | c.6938 N (Acceptor Site) | N | 0.997496 | 0.997267 | 0.000229 | 0.00 | 83.29 | 83.29 | 0.8329 | 5.54 | 5.54 | 0.29 | 0.4 | 0.4 | 0.1058063 | ND |  | ND | 0 | 0.02686 |
| 35 | BRCA2 | c.7008-44A>G | c.7008 N (Acceptor Site) | N | 0.995047 | 0.99538 | 0.000333 | 0.00 | 82.03 | 82.03 | 0.8203 | 10.37 | 10.37 | 0.60 | 0.56 | 0.56 | 0.04 | 4.45 | 4.68 | 0.21 | 0 | 0.02686 |
| 36 | BRCA2 | c.7008-62A>G | c.7008 N (Acceptor Site) | N | 0.995047 | 0.994974 | 7.3E-05 | 0.00 | 82.03 | 82.03 | 0.8203 | 10.37 | 10.37 | 0.60 | 0.56 | 0.56 | 0.04 | 4.45 | 4.26 | 0.21 | 0 | 0.02686 |
| 37 | BRCA2 | c.7806-15delC | c.7806 N (Acceptor Site) | N | 0.987322 | 0.990172 | 0.00285 | 0.00 | 98.45 | 98.45 | 0.9845 | 8.33 | 7.80 | 0.46 | 0.95 | 0.88 | 0.49 | 3.73 | 2.03 | 0.15 | 0 | 0.04424 |
| 38 | BRCA2 | c.7806-40A>G | c.7806 N (Acceptor Site) | N | 0.987322 | 0.98583 | 0.001492 | 0.00 | 98.45 | 98.45 | 0.9845 | 8.33 | 8.33 | 0.46 | 0.95 | 0.95 | 0.49 | 3.73 | 3.8 | 0.15 | 0 | 0.02686 |
| 39 | BRCA2 | c.8332-54T>G | c.8332 N (Acceptor Site) | N | 0.997311 | 0.996628 | 0.000683 | 0.00 | 82.39 | 82.39 | 0.8239 | 4.26 | 4.26 | 0.21 | 0.91 | 0.91 | 0.70 | ND |  | ND | 0 | 0.02686 |
| 40 | BRCA2 | c.8633-16C>G | c.8633 N (Acceptor Site) | N | 0.999279 | 0.999331 | 5.2E-05 | 0.00 | 87.66 | 87.66 | 0.8766 | 6.97 | 6.97 | 0.38 | 0.99 | 0.98 | 0.61 | 6.25 | 5.64 | 0.27 | 0 | 0.02686 |
| 41 | BRCA2 | c.9257-16T>C | c.9257 N (Acceptor Site) | N | 0.99451 | 0.993835 | 0.000675 | 0.00 | 91.58 | 91.58 | 0.9158 | 13.32 | 13.46 | 0.78 | 0.98 | 0.96 | 0.20 | 10.29 | 10.22 | 0.48 | 0 | 0.02459 |
| 42 | BRCA2 | c.9257-113T>G | c.9257 N (Acceptor Site) | N | 0.99451 | 0.994584 | 7.4E-05 | 0.00 | 91.58 | 91.58 | 91.58 | 13.32 | 13.32 | 4.89 | 0.98 | 0.98 | 3.91125 | 10.29 | 10.29 | 0.30 | 0 | 0.02686 |
| 43 | BRCA2 | c.9257-83G>A | c.9257 N (Acceptor Site) | N | 0.99451 | 0.995001 | 0.000491 | 0.00 | 91.58 | 91.58 | 0.9158 | 13.32 | 13.32 | 0.78 | 0.98 | 0.98 | 0.20 | 10.29 | 10.29 | 0.48 | 0 | 0.02686 |
| 44 | BRCA2 | c.9649-20C>T | c.9649 N (Acceptor Site) | N | 0.937437 | 0.965023 | 0.027586 | 0.01 | 91.32 | 91.32 | 0.9131 | 8.54 | 8.80 | 0.48 | 0.96 | 0.97 | 0.48 | 3.26 | 2.76 | 0.13 | 0 | 0.02095 |
| 45 | BRCA2 | c.7008-44A>T | c.7008 N (Acceptor Site) | N | 0.995047 | 0.994921 | 0.000126 | 0.00 | 82.03 | 82.03 | 0.8203 | 10.37 | 10.37 | 0.60 | 0.56 | 0.56 | 0.04 | 4.45 | 4.48 | 0.21 | 0 | 0.02686 |
| 46 | BRCA2 | c.9649- 65_9649- 62delACTT | c.9649 N (Acceptor Site) | N | 0.937437 | 0.927514 | 0.009923 | 0.00 | 91.32 | 91.32 | 0.9132 | 8.54 | 8.54 | 0.48 | 0.96 | 0.96 | 0.48 | 3.26 | 2.91 | 0.13 | 0 | 0.02686 |
| 47 | BRCA2 | c.425+33A>G | c.425 N (Donor Site) | N | 0.958914 | 0.954665 | 0.004249 | 0.00 | 84.19 | 84.19 | 0.8419 | 9.11 | 9.11 | 0.52 | 0.94 | 0.94 | 0.42 | 0.88 | 1.02 | 0.02 | 0 | 0.02686 |
| 48 | BRCA2 | c.475+26T>C | c.475 N (Donor Site) | N | 0.740802 | 0.699582 | 0.04122 | 0.01 | 84.50 | 84.50 | 0.8449 | 9.46 | 9.46 | 0.54 | 0.95 | 0.95 | 0.41 | ND |  | ND | 0 | 0.02686 |
| 49 | BRCA2 | c.516+14C>T | c.516 N (Donor Site) | N | 0.513504 | 0.563115 | 0.049611 | 0.00 | 87.54 | 87.54 | 0.8754 | 8.88 | 8.88 | 0.50 | 0.98 | 0.98 | 0.48 | ND |  | ND | 0 | 0.02686 |
| 50 | BRCA2 | c.516+21A>T | c.516 N (Donor Site) | N | 0.513504 | 0.535726 | 0.022222 | 0.00 | 87.54 | 87.54 | 0.8754 | 8.88 | 8.88 | 0.50 | 0.98 | 0.98 | 0.48 | ND |  | ND | 0 | 0.02686 |
| 51 | BRCA2 | c.631+25C>T | c.631 N (Donor Site) | N | 0.880385 | 0.907043 | 0.026658 | 0.00 | 78.15 | 78.15 | 0.7815 | 6.84 | 6.84 | 0.38 | 0.44 | 0.44 | 0.06 | ND |  | ND | 0 | 0.02686 |
| 52 | BRCA2 | c.793+46G>A | c.793 N (Donor Site) | N | 0.99801 | 0.998043 | 3.3E-05 | 0.00 | 91.18 | 91.18 | 0.9118 | 11.01 | 11.01 | 0.63 | 1.00 | 1.00 | 0.37 | 5.95 | 6.22 | 0.23 | 0 | 0.02686 |
| 53 | BRCA2 | c.6937+74C>G | c.6937 N (Donor Site) | N | 0.8554 | 0.860679 | 0.005279 | 0.00 | 73.70 | 73.70 | 0.737 | 4.79 | 4.79 | 0.25 | 0.64 | 0.64 | 0.39 | ND |  | ND | 0 | 0.02686 |
| 54 | BRCA2 | c.7007+34A>G | c.7007 N (Donor Site) | N | 0.952109 | 0.956547 | 0.004438 | 0.00 | 81.63 | 81.63 | 0.8163 | 10.53 | 10.53 | 0.61 | 0.99 | 0.99 | 0.38 | ND |  | ND | 0 | 0.02686 |
| 55 | BRCA2 | c.7435+14A>G | c.7435 N (Donor Site) | N | 0.58766 | 0.492267 | 0.095393 | 0.00 | 68.29 | 68.29 | 68.29 | 5.64 | 5.64 | 3.92 | 0.25 | 0.25 | 3.665625 | ND |  | ND | 0 | 0.02686 |
| 56 | BRCA2 | c.7617+14_761 7+15delTA | c.7617 N (Donor Site) | N | 0.988975 | 0.991735 | 0.00276 | 0.00 | 89.83 | 89.83 | 0.8983 | 9.80 | 9.80 | 0.56 | 0.99 | 0.99 | 0.43 | 7.41 | 6.42 | 0.29 | 0 | 0.02686 |
| 57 | BRCA2 | c.7976+23C>T | c.7976 N (Donor Site) | N | 0 | 0 | 0 | 0.00 | 100.00 | 100.00 | 1 | ND |  | ND | ND |  | ND | ND |  | ND | 0 | 0.02686 |
| 58 | BRCA2 | c.7976+35C>A | c.7976 N (Donor Site) | N | 0 | 0 | 0 | 0.00 | 100.00 | 100.00 | 1 | ND |  | ND | ND |  | ND | ND |  | ND | 0 | 0.02686 |
| 59 | BRCA2 | c.8331+22G>C | c.8331 N (Donor Site) | N | 0.980171 | 0.975246 | 0.004925 | 0.01 | 87.54 | 87.54 | 0.8753 | 8.88 | 8.88 | 0.50 | 0.96 | 0.96 | 0.46 | 2.27 | 2.22 | 0.08 | 0 | 0.02686 |
| 60 | BRCA2 | c.8487+104G> A | c.8487 N (Donor Site) | N | 0.907752 | 0.907746 | 6E-06 | 0.00 | 84.50 | 84.50 | 84.5 | 9.46 | 9.46 | 4.69 | 0.95 | 0.95 | 3.74 | ND |  | ND | 0 | 0.02686 |
| 61 | BRCA2 | c.8487+81C>T | c.8487 N (Donor Site) | N | 0.907752 | 0.918162 | 0.01041 | 0.00 | 84.50 | 84.50 | 0.845 | 9.46 | 9.46 | 0.54 | 0.95 | 0.95 | 0.41 | ND |  | ND | 0 | 0.02686 |
| 62 | BRCA2 | c.8953+98T>C | c.8953 N (Donor Site) | N | 0.999788 | 0.999772 | 1.6E-05 | 0.00 | 100.00 | 100.00 | 1 | 10.86 | 10.86 | 0.62 | 1.00 | 1.00 | 0.38 | 4.24 | 4.24 | 0.16 | 0 | 0.02686 |
| 63 | BRCA2 | c.9256+58A>T | c.9256 N (Donor Site) | N | 0.997744 | 0.997853 | 0.000109 | 0.00 | 82.59 | 82.59 | 0.8259 | 9.43 | 9.43 | 0.54 | 0.95 | 0.95 | 0.41 | ND |  | ND | 0 | 0.02686 |
| 64 | BRCA2 | c.9501+9A>C | c.9501 N (Donor Site) | N | 0.951684 | 0.951896 | 0.000212 | 0.00 | 91.93 | 91.93 | 0.9193 | 10.28 | 10.28 | 0.59 | 0.99 | 0.99 | 0.40 | 1.64 | 2.82 | 0.05 | 0 | 0.02686 |
| ND: Not detected. | | | | | | | | | | | | | | | | | | | | | | |

| **Table S2.** Missing rates of the eight in silico tools | | |  |
| --- | --- | --- | --- |
| In silico tools | No. of missing scores  (positive/ negative variants) | Missing rate (%)  (No. of missing scores/ No. of total examined) | |
| SpliceRover | 4 (4/0) | 2.3 (4/174) | |
| SpliceAI | 1 (1/0) | 0.6 (1/174) | |
| SSF | 0 (0/0) | 0 (0/174) | |
| MES | 2 (0/2) | 1.2 (2/174) | |
| NNSplice | 3 (12) | 1.7 (3/174) | |
| GeneSplicer | 43 (24/19) | 24.7 (43/174) | |
| HSF | 12 (12/0) | 6.9 (12/174) | |
| SPiCE | 3 (3/0) | 1.7 (3/174) | |
